# Hierarchically Structured Stimuli-Responsive Liquid Crystalline Terpolymer–Rhodamine Dye Conjugates

**DOI:** 10.3390/molecules30020401

**Published:** 2025-01-18

**Authors:** Samiksha Vaidya, Meenakshi Sharma, Christian Brückner, Rajeswari M. Kasi

**Affiliations:** 1 Department of Chemistry, University of Connecticut, Storrs, CT 06269, USA; vaidyasamiksha93@gmail.com (S.V.); meenakshi.sharma@uconn.edu (M.S.); 2 Polymer Program, Institute of Material Science, University of Connecticut, Storrs, CT 06269, USA

**Keywords:** terpolymers, liquid crystal polymers, stimuli-responsive materials, T-dependent SAXS

## Abstract

Optically responsive materials are applied in sensing, actuators, and optical devices. One such class of material is dye-doped liquid crystal polymers that self-assemble into cholesteric mesophases that reflect visible light. We report here the synthesis and characterization of a family of linear and mildly crosslinked terpolymers prepared by the ROMP of norbornene-based monomers. The three monomers were composed of (i) rhodamine dye through one or two norbornene end groups utilizing flexible C_10_-alkane spacers, (ii) a cholesteryl liquid crystal (LC) using C_9_-alkane spacers, and (iii) PEG side chains. We investigated how these architectural variations in these terpolymers impacted their hierarchically self-assembled mesophase properties. We probed their composition, morphology, thermal, mechanic, photochromic, and mechanochromic properties using, inter alia, ^1^H NMR spectroscopy, DSC, temperature-dependent SAXS, diffuse reflectance UV-vis spectroscopy, and optical polarization microscopy. The new terpolymers exhibited architecture-dependent thermochromic, mechanochromic, and piezochromic properties arising from LC–rhodamine dye interactions. We found that a compromise between the rigidity and flexibility of the terpolymer architectures needed to be stricken to fully express stimuli-responsive properties. These terpolymers also showed distinctly different properties compared to those of a previously reported structurally related liquid crystalline copolymer made from two monomers. These findings help to define the design principles for optimally stimuli-responsive liquid crystalline polymers.

## 1. Introduction

Hybrid materials comprising LC polymer frameworks and dye molecules offer new understanding into how the dyes impact the electro-optical, morphological, thermal, and mechanical properties and applications of liquid crystalline polymers (LCPs). Specifically, there is a lot of interest in dye-doped LCPs and dyes covalently bound with LC frameworks that self-assemble into cholesteric mesophases suited to reflect light in the range of 350–700 nm; this is the basis for their utility as lasing materials, optical materials, and sensors [1,2,3,4,5,6,7]. However, there is a need to broaden or shift the wavelengths of light reflected in the near IR region [8] to design materials for optical lasing applications [9,10], optically responsive actuating materials [11,12], luminescent solar concentrators [13], broadband reflection materials [7,14], infrared regulating smart windows [15,16,17], and chemosensors [18].

Doping LCPs with organic dyes offer a simple approach to introduce optical functionality [16,19,20]. Some of the dye molecules that have been used include rhodamines and azobenzenes [21]. Rhodamine chromophore was chosen due to its excellent optical properties (large extinction coefficient and high-fluorescence quantum yield), high chemical stability, ability to functionalize, and demonstrated ability to form stimuli-responsive materials [22]. The *cis-trans* isomerization of azobenzene dyes has been conducted to optically actuate liquid crystalline polymers with light [14,23,24,25]. However, doping may impart changes to the liquid crystalline mesomorphic structures [26,27]. Further impediments for the realization of dye-doped LCP systems are the possible limited solubility of dye molecules in the LCP matrix, restricting dye loading [27]. Secondly, maintaining a specific dye orientation in fairly low T_g_ (<50 °C) LCP hosts is challenging [27,28]. Some of these drawbacks can be overcome by covalently attaching a non-mesogenic dye to the polymer backbone. This may result in a considerable increase in dye content within the LC phases, without loss of the mesophase character, or leading to phase separation [27,29]. Covalently linking dyes to LCPs also proved to, e.g., accelerate the *cis*-to-*trans* isomerization kinetics of some azo dyes compared to the kinetics of dyes doped in a host polymer matrix, whereby factors such as chain flexibility were crucial for the rate of the thermal isomerization process [30]. Also, an azobenzene dye incorporated into a partially crosslinked system that formed a cholesteric LC created a pitch gradient upon trans-cis isomerization, leading to a large change in its optical properties [14]. In another example, rhodamine-based acrylates were crosslinked with cholesteric mesophase-forming liquid crystalline monomers; the self-assembled cholesteric liquid crystalline elastomer showed both mechanochromic and chemochromic behaviors due to the stimuli-responsive spiropyran unit within rhodamine.

In this work, a rhodamine derivative [31] was covalently linked with different terpolymers that self-assemble into LCPs. By choosing rhodamine derivatives that carried one or two polymerizable end groups (norbornene) and by varying the tether length between the dye and the end group, we were able to realize linear and mildly crosslinked dye-containing, random terpolymers. We present a systematic study of the impact of the nature of these terpolymers on their morphology and hierarchical self-assembly. The corresponding thermal, mechanic, photochromic, and mechanochromic property data are presented, with a focus on the elucidation of the modulation of the LC mesophase evolution and LC–dye interactions. Insight into these functional polymers will assist in the realization of ’smart polymers’ in the fields of mechanical damage sensing, actuators, and novel photonic materials.

## 2. Results and Discussion

### 2.1. Synthesis of the Terpolymers

**Scheme 1:**
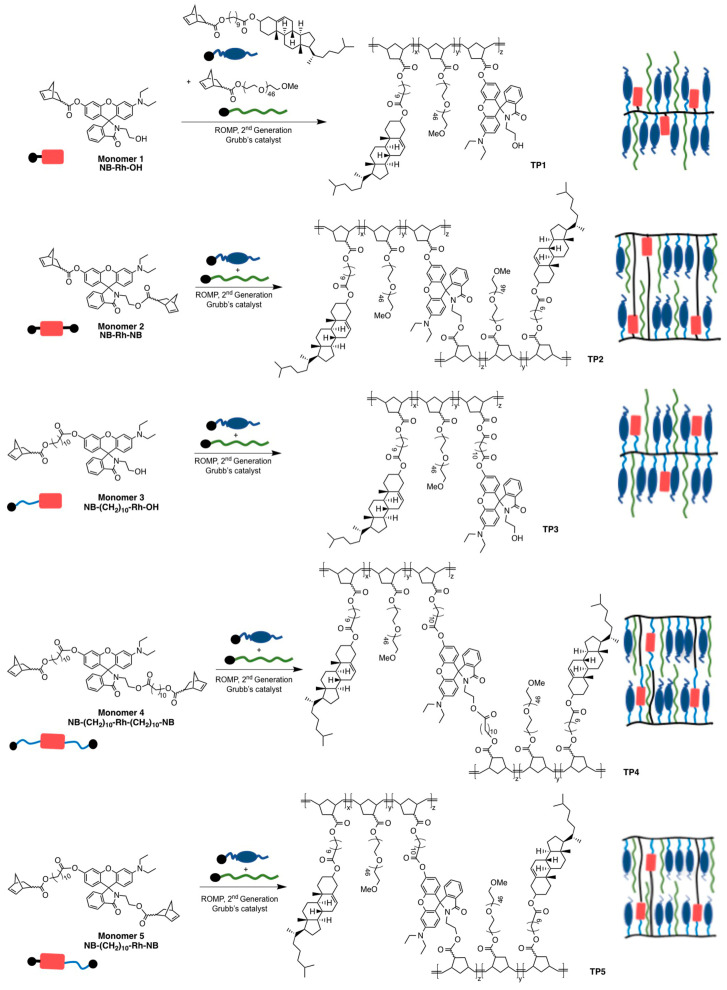
Synthesis of terpolymers **TP1** through **TP5**, including cartoon representations of their monomers and terpolymer structures. Rhodamine dyes are red rectangles. Cholesteryl liquid crystals are blue cylinders. PEG with spacers are green wavy lines. Norbornene units are black dots attached to rhodamines, cholesteryl LC or PEG units with or without spacers.

Four terpolymers **TP1**–**TP5** were synthesized by ruthenium-catalyzed ring-opening metathesis polymerization (ROMP) using five different dye monomers, **Monomers 1**–**4** reported previously [32], and the novel **Monomer 5**, together with two other known co-monomers: the side-chain liquid crystalline monomer **NBCh9 [33]** and the side-chain PEG-extended monomer **NBMPEG [1]** (Scheme 1). The hybrid **Monomer 5** was designed to link on one side to norbornene via a 10-carbon methylene spacer, and the other side linked directly to norbornene. As detailed below, the spacer was established to assist in the formation of a cholesteric phase and direct linkage to help in its mechanochromic behavior. Details of the synthesis of **Monomer 5** are provided in the ESI.

Terpolymers **TP1** through **TP5** resulting from the ROMP of the mixture of the three monomers are statistically random copolymers of the compositions and physical properties listed in Table 1. Terpolymers **TP1** and **TP3** are linear, while **TP2**, **TP4**, and **TP5** containing bifunctional monomers are mildly crosslinked.

The controls that were prepared included a random copolymer **CP** of ~20 kDa comprising ∼85 wt % of **NBCh9** and ∼15 wt % of **NBMPEG** synthesized based on a previously reported protocol [1] and **CP+HO-Rh-OH**, a physical blend of **CP** and **HO-Rh-OH** prepared by mixing **CP** with **HO-Rh-OH** in the same ratio as present in the terpolymers.

### 2.2. Thermal Properties of the Terpolymers

Terpolymers **TP1**–**TP4** proved to be thermally stable up to 325–350 °C, as determined by TGA (Figure S8). The first cooling DSC curves of **TP1** and **TP2** showed glass transitions (T_g_) in addition to one mesophase transition, which coincided with the mesophase isotropization or the liquid crystalline clearing temperature (Table 1). However, terpolymers **TP3**–**TP5** showed a broad T_g_ with a mesophase transition and a separate mesophase clearing temperature. For reproductions of the first cooling DSC curves of all the terpolymers, see the ESI.

We interpret the findings in relation to the architecture of the terpolymers. The presence of methylene spacers between norbornene and rhodamine in **TP3** and **TP4** imparted sufficient motional decoupling, such that T_g_ for these terpolymers was slightly lower than those values for **TP1** and **TP2,** linking the norbornene moieties directly to rhodamine. All the terpolymers that contain **NBMPEG** as a side-chain monomer showed PEG recrystallization around −37 °C (*T*_c_). We understand this depression of *T*_c_ to be due to a nanoconfinement of the PEG domains, as previously observed in LCRBCs and microphase-segregated BCPs^1^. The mesophases and their corresponding transition temperatures were identified in temperature-controlled small-angle X-ray scattering experiments, UV visibility studies, and polarized optical microscopy images presented in the dedicated chapters below.

### 2.3. Temperature-Dependent Small-Angle X-Ray Scattering Investigations of the Terpolymers

The mesophase evolution and hierarchical structures of all the terpolymers were studied using room temperature and temperature-controlled small-angle X-ray scattering (T-SAXS). The room temperature SAXS data of all the terpolymers and their corresponding d spacings are presented in the ESI. Terpolymers **TP1** and **TP2** self-assembled into microphase-segregated PEG domains (q* peak at ~0.06 Å, ~10–16 nm domains) that lack long-range order [1,34]. This phase co-existed with interdigitated smectic A (SmA_d_) mesophases between room temperature and ~83 °C (for illustrated reproductions of the SAXS data of **TP1** and **TP2**, see the ESI). Above ~83 °C, the SmA_d_ mesophases transitioned into their isotropic states with the retention of the PEG microphase-segregated domains. The hierarchical structures and mesophase properties of **TP1** and **TP2** were comparable with each other despite the existence of crosslinked networks within **TP2**, though crosslinking increased the q* domain size and broadened the PEG domains. The cholesteryl side-chain length was calculated to be 3.34 nm, assuming a fully extended structure [35]. The molecular lengths of **Monomers 1** and **2** were calculated to be 1.65 and 2.20 nm, respectively. The d_1_ value for **TP1** and **TP2** was determined to be 5.23 nm, which is less than ½ of cholesteryl side-chain length of 3.34 nm, consistent with the interdigitated smectic domains of Ch9 molecules (cholesteryl with C_9_ spacer) interspersed with the dye bearing **Monomers 1** and **2**, respectively.

Terpolymer **TP3** showed a q* peak at 0.06 Å, indicating disordered microphase segregated PEG domains of ~10–16 nm size [1] and *q*_LC1_, *q*_LC2_, and *q*_LC3_, representing LC ordering and the co-existence of smectic phases at room temperature (see the ESI for a schematic interpretation of the DSC and SAXS data for **TP3**). The intensity of smectic layers gradually increased up to 67.6 °C (mesophase transition temperature, *T*_1_). Above 67.6 °C, a small amount of smectic mesophase was retained, and the mesophases finally disappeared above ~89.4 °C (mesophase transition temperature *T*_2_ or *T*_CL_) in agreement with DSC. The mesophases could be cycled by cooling from the isotropic phase. Additionally, the shift in the *q*_LC1_ peak indicated the presence of SmC and was also supported by the d-spacing values of d_1_ (*q*_LC1_) = 6.3 nm, d_2_ (*q*_LC3_) = 3.6 nm, and d_3_ (*q*_LC3_) = 2.3 nm. The d-spacing value of d_2_ suggested a smectic A monolayer (SmA_1_), demonstrating the coexistence of different smectic mesophases (smectic polymorphism) of the Ch9 (~3.34 nm) domains mixed in with Monomer 3 (~3.3 nm). The coexistence of a cholesteric phase along with some enchained smectic domains beyond first thermal transition *T*_1_ was supported by the UV-vis reflectance data (see Figure S13 and the POM analysis results, Figure S15).

For terpolymer **TP4**, q* corresponded to the microphase segregation of PEG side chains; *q*_LC1_ and *q*_LC2_ corresponded to the reflections from LC ordering in the 1D T-SAXS data at room temperature. See the ESI for a schematic interpretation of DSC and T-SAXS for **TP4**. It was observed that the q* domain size was slightly lower as compared to the non-spacer analog **TP2**. This was also observed when comparing the behaviors of **TP1** and **TP3**. The smectic mesophases were stable up to 60.4 °C. By comparing the d-spacing values of d_1_ (*q*_LC1_) = 5.23 nm and d_2_ (*q*_LC2_) = 3.5 nm with the side-chain monomer length of Ch9 (~3.34 nm), the coexistence of a smectic A interdigitated mesophase (SmA_d_) and a smectic A monolayer (SmA_1_) was determined. Unlike **TP3**, the LC order reflections were not sharp or distinct; also, the co-existing smectic mesophases did not seem to transition well into another mesophase above 60.4 °C. This may be due to the bulkiness of monomer 4 (~4.5 nm) compared to Ch9 (~3.34 nm), as well as the higher-than-expected incorporated total of **Monomer 4** (Table 1). The mesophases cleared above the isotropization temperature (*T*_2_ ~74.2 °C), similar to the thermal transition data derived from DSC.

**Figure 1:**
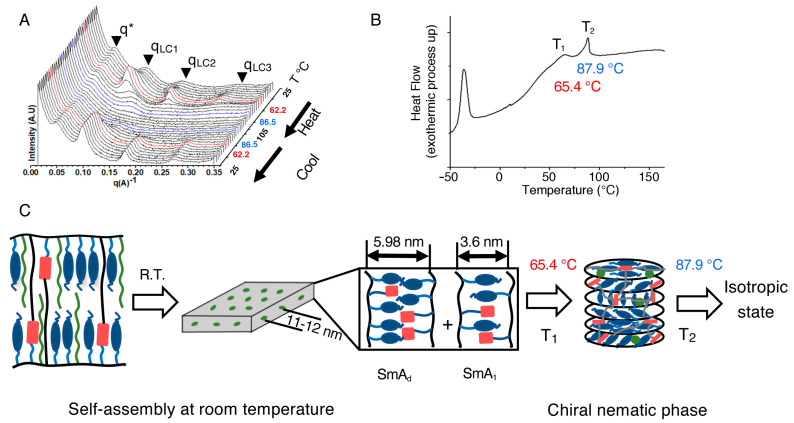
(**A**) 2-Dimensional data derived from T-SAXS scan of **TP5**; * indicates primary peak that is correlated to PEG domains. (**B**) First cooling DSC cycle of **TP5**. (**C**) Cartoon representation of phase evolution of **TP5** during heating cycle. In the schematic, rhodamine dyes are represented as red rectangles, cholesteryl liquid crystals as blue cylinders, PEG units as green wavy lines, and norbornenes as black dots with or without spacers.

The mesostructural study of **TP5** showed distinct and clear mesophase evolution, as per the T-SAXS data (Figure 1). The one-dimensional T-SAXS data for TP5 showed a q* peak at 0.06 Å, indicating microphase-segregated PEG domains of ~11–12 nm and *q*_LC1_, *q*_LC2_, and *q*_LC3_, representing LC ordering and the co-existence of smectic phases (SmA_d_ and SmA_1_ phases) at room temperature. The intensity of smectic layers gradually increased up to ~65.4 °C (mesophase transition temperature, *T*_1_). Above 65.4 °C, the smectic mesophase may have been retained in addition to cholesteric or chiral nematic mesophases that finally disappeared above 86.5 °C (mesophase transition temperature *T*_2_ or *T*_CL_) in agreement with DSC. The mesophases could be cycled by cooling from the isotropic phase. The d-spacing value of d_1_ = 5.98 nm suggested the interdigitated smectic layer SmA_d_. The d-spacing value of d_2_ = 3.6 nm suggested the smectic A monolayer (SmA_1_), demonstrating the coexistence of different smectic mesophases (smectic polymorphism) (Figure S10). The presence of smectic mesophases from the Ch9 units (~3.34 nm) mixed in with **Monomer 5** (~3.9 nm) in **TP5** agreed well with T-SAXS fitting, as well as the molecular lengths of Ch9 and **Monomer 5**. The coexistence of the cholesteric phase along with some enchained smectic domains beyond the first thermal transition *T*_1_ was further supported by the UV-vis reflectance POM analysis results discussed below.

### 2.4. Polarized Optical Microscopy of the Terpolymers Films

Terpolymers **TP1** through **TP5**, as well as the control samples, were compression-molded using a carver press into free-standing films. The processing temperature for each sample was set using the transition temperatures determined by DSC. By compression-molding the terpolymers above the clearing temperature, the smectic or cholesteric mesophases were retained.

**Figure 2:**
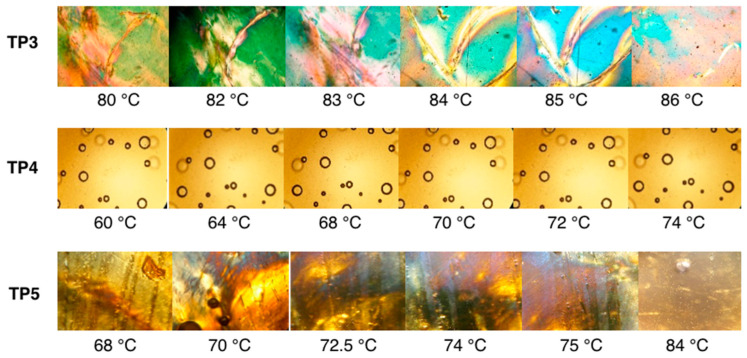
Polarized optical microscopy (POM) images of terpolymers **TP3**, **TP4**, and **TP5** at temperatures indicated.

The textures of **TP1** through **TP5** were individually observed under a polarized optical microscope (POM) equipped with a hot stage (Figure 2; for images of all the terpolymers, see Figure S15). **TP1**, **TP2,** and **TP4** did not exhibit any oily textures at the temperatures indicated, while **TP3** and **TP5** showed oily textures. Consequently, only **TP3** and **TP5** when processed above the clearing temperature and imaged around their respective transition temperature T2 showed the presence of cholesteric textures.

### 2.5. UV-Vis Reflectance Spectroscopy of the Terpolymers Films

**Figure 3:**
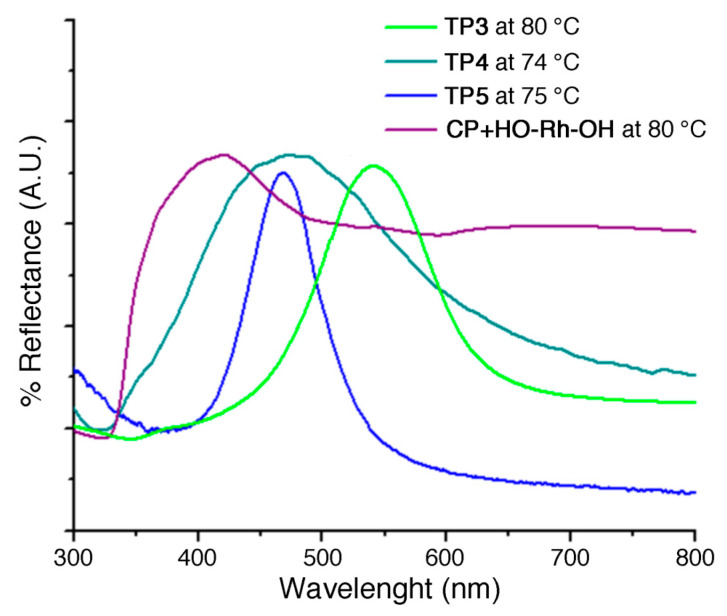
The UV-vis diffuse reflectance spectra of the terpolymers at the temperatures indicated. For the corresponding spectra of all the other terpolymers and the reference materials, see the ESI.

The optical properties of the compression-molded samples were evaluated by UV-vis diffuse reflectance spectroscopy. The film of copolymer **CP** is violet; its reflectance spectrum showed a distinct band at λ_max_ = 410 nm (for the UV-vis reflectance spectra of all the samples, see the ESI). The compression-molded film of rhodamine **HO-Rh-OH** molded at 80 °C appeared to range from off-white to brown, indicating that rhodamine is present in its ring-closed achromatic structure. In contrast, a film of rhodamine **HO-Rh-OH** molded at 215 °C possessed the characteristic bright pink color of the ring-opened form of rhodamine [32]. The blend **CP+HO-Rh-OH** compression-molded at 80 °C resulted in a violet-colored film, with white spots of **HO-Rh-OH** physically dispersed in the film. Its reflectance spectrum shows the corresponding peak at 410 nm (Figure 3), albeit it is much broader when compared to the spectrum of the film of the pure copolymer **CP**.

**Figure 4:**
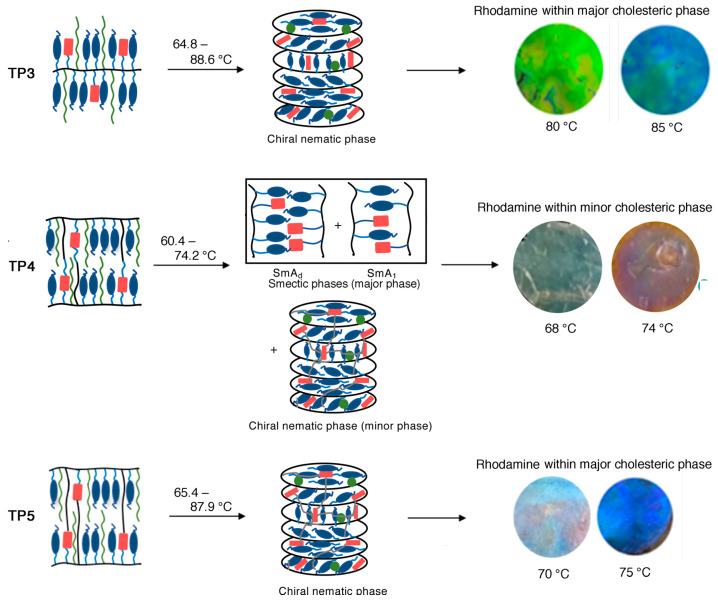
Photographic images of the colors of the compression-molded films of **TP3** through **TP5** prepared using a carver press at the temperatures indicated and a graphical representation of the phase models rationalizing the colors observed. In the schematic, rhodamine dyes are represented as red rectangles, cholesteryl liquid crystals as blue cylinders, and PEGs as green wavy lines.

When compression-molded at 80 °C, a temperature at or slightly above T1, the film of **TP1** appeared to range from orange to brown with a broad reflectance spectrum, with λ_max_ at λ610 nm (Figure 4). Under similar conditions, **TP2** delivered a dark brown-black film with no discernable reflection signal. **TP3** compression-molded at 80 °C, i.e., well above T1 (67.6 °C), but below T2 (89.4 °C), had a bright green film, with a strong reflectance peak at λ_max_ = 550 nm (Figure 3 and Figure 4). The same material compression-molded at 85 °C changed to blue, suggesting that the cholesteric pitch length had shortened upon the increase in temperature; this was also confirmed by POM. The films of **TP4** processed between 60 and 80 °C presented faint green- and light violet-colored reflections at 68 °C and 74 °C. The UV-vis reflectance spectra of this film showed a very broad reflection peak around λ_max_ = 460 nm. Combined with the fact that a minimal cholesteric oily streak was observed in its POM image, this suggests that the cholesteric mesophase is a minor component within the smectic system (Figure 3 and Figure 4).

Terpolymer **TP5** was compression-molded at 70.0 °C, again a temperature above the first thermal transition T1 (65.4 °C), but below T2 (87.9 °C). The resulting film showed a bright blue and uniform color (Figure 4) with a sharp and narrower reflectance peak at 460 nm, suggestive of the formation of a well-defined cholesteric phase; the latter finding was commensurate with the presence of an oily texture in its POM micrographs that also cleared around at the LC clearing temperature (at 87.9 °C). The reflectance color of **TP5** is bright blue. This material does not reflect a broader color range like **TP3** that shows bright green and blue colors. This is possibly due the crosslinked architecture of **TP5** that restricts the cholesteric structure. When comparing the colors of **TP5** and **CP+HO-Rh-OH**, the red-shifted reflected color from **TP5** confirms the cooperative interaction of the ring-closed form of **HO-Rh-OH** with the cholesteric phase of **TP5**.

We interpret the differences seen in the optical properties of the compression-molded films of terpolymers **TP1** through **TP5** as being mainly due to the differing connectivities of the **HO-Rh-OH** monomers. **TP1** is a linear terpolymer with ring-closed rhodamines (11.1 wt%) that does not favor cholesteric mesophase formation and primarily self-assembled into a smectic mesophase coexisting with non-photonic smectic mesophases. **TP2** is a partially crosslinked terpolymer without a spacer between the chromophore and the polymer backbone. This rendered the polymer backbone rigid, and only the dark brown/blackish colors of non-photonic smectic mesophases and ring-closed rhodamines (30.2 wt%) were observed. In contrast, the linear terpolymer **TP3** is, on account of the 10-carbon methylene spacers between the chromophore (8.8 wt%) and the polymer backbone, more flexible. It thus was capable of forming a bright green cholesteric phase. The thermochromic properties in **TP3** arise primarily from cooperative interaction between the 1D photonic Ch* mesophase that is co-enchained with ring-closed rhodamine chromophores. In the crosslinked terpolymer **TP4**, the rhodamine chromophore (26.9 wt%) is linked to the polymer backbone via two 10-carbon methylene spacers on either side. A broad cholesteric reflection was observed due to the mobility and flexibility of the architecture and is a minor component of smectic mesophases. Finally, **TP5** demonstrates, structurally, a compromise between the flexibility provided by the presence of one 10-carbon methylene spacer between the chromophore (21.3 wt%) and the polymer backbone and the rigidity provided by the single direct linkage within this crosslinked architecture. It is thusly ideally set up to form a well-developed cholesteric phase with some enchained smectic domains beyond the first thermal transition *T*_1_.

Thus, in each terpolymer, dye enchainment is statistically random without crosslinks (TP1 and TP3), or it is statistically random and crosslinked (TP2, TP4, and TP5). Using detailed small-angle X-ray analysis and optical microscopy, we have established how the dye prior to mechanical stress interacts with the liquid crystalline polymer or the liquid crystalline network. Terpolymers TP3 through TP5, which contain spacers between rhodamine and the norbornene backbone, form self-assembled dyes containing a cholesteric mesophase (Figure 4). The importance of the crosslinked networks is discussed below.

### 2.6. Mechanochromic or Piezochromic Properties of the Terpolymers

#### 2.6.1. Effect of Mechanical Abrasion

**Figure 5:**
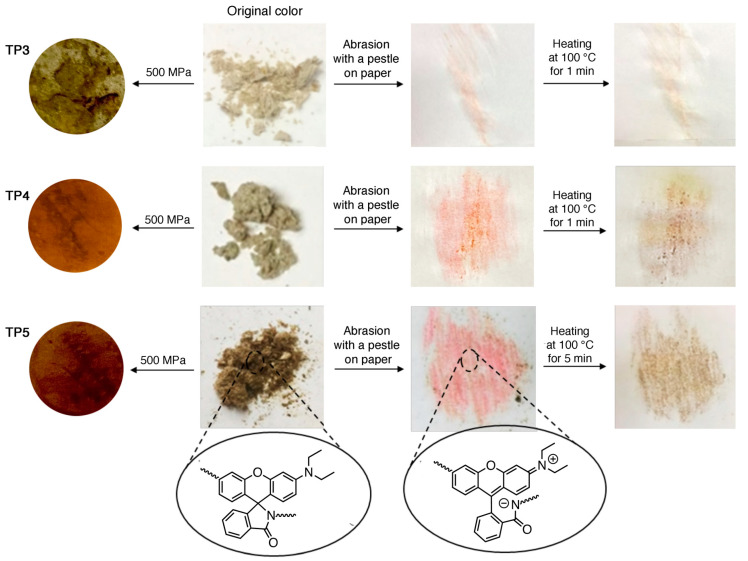
Photographic images of the color changes observed in the compression experiments and the thermally reversible abrasion experiments using terpolymers **TP3** through **TP5**. The deficient piezochromic responses of terpolymers **TP1** and **TP2** are shown in the ESI.

To test the mechanochromic behavior of terpolymers **TP1** through **TP5**, powdered samples were abraded on paper using a porcelain pestle. The resulting abrasion-induced color changes were noted for each terpolymer (Figure 5 and Figure S15).

The samples of the linear terpolymer **TP1** and its methylene spacer-modified analogue **TP3** did not show significant color change upon abrasion (Figure S15). In contrast, the crosslinked terpolymer **TP2** and its spacer-modified analogue **TP4** changed from whitish grey to orangish red and faint pinkish red, respectively, while terpolymer **TP5** changed from a brown color to dark red (Figure 5 and Figure S15).

**Figure 6:**
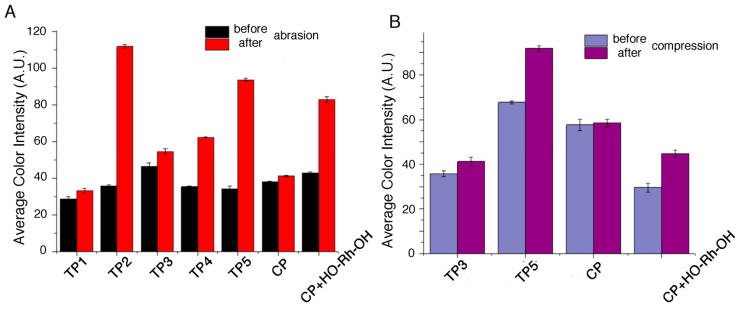
(**A**) Color intensities of terpolymers **TP1** through **TP5** and two control samples **CP** and **CP+HO-Rh-OH** before and after abrasion treatment. (**B**) Color intensities of terpolymers **TP3** and **TP5** and two control samples **CP** and **CP+HO-Rh-OH** before and after compression at 500 MPa. Both were analyzed by ImageJ software, URL https://imagej.net/ij/ accessed on 5 January 2025. Raw histogram data are shown in ESI.

The color change in the rhodamine-modified polymers originates from the mechanochemical isomerization of a certain fraction of the spirolactam leuco-forms to the planarized ring-opened zwitterionic chromophores [19]. The color intensity provides a relative measure of the fraction of opened rings. This, in turn, is a measure of how efficiently the mechanical stimulus is translated through the polymer into the ring-opening reaction. To quantify the relative color intensities, the images of the polymers before and after abrasion were analyzed using Image-J (https://imagej.net/ij/, URL accessed on 5 January 2025, Figure 6A). This analysis confirmed that polymer **TP2** showed better mechanochromic features than polymer **TP4**, while hybrid polymer **TP5** exhibited the strongest response overall (Figure 6A).

#### 2.6.2. Effect of Hydrostatic Compression

The piezochromic properties of terpolymer powders of samples **TP1** through **TP5** (and control sample **CP** and control sample **CP+HO-Rh-OH**) were tested by pressing them in a pellet-pressing die in a Carver press at 500 MPa. This value was chosen as it was previously shown that the ring-opening reaction of the spirolactam is maximized at this pressure [32]. The polymer films thus formed were removed from the die, and their colors were recorded (Figure 5 and Figure S15).

To quantify the relative color intensities, the images of the polymers before and after abrasion were analyzed using Image-J (URL https://imagej.net/ij/ accessed on 5 January 2025, Figure 6A). Under these conditions, control sample **CP** did not exhibit any change in color (Figure S15), nor did linear polymers **TP1** and **TP3** (Figure 6B and Figure S15). However, the control sample mixture **CP+HO-Rh-OH** and the crosslinked terpolymers **TP2** (Figure S15), **TP4**, and **TP5** (Figure 5) showed a piezochromic response. Polymer **TP2** exhibited a dull orangish-brown colored film, **TP4** showed a reddish-orange film, and **TP5** produced a dark reddish film; their color densities were quantified (Figure 6B). The piezochromic results were qualitatively in agreement with the trends of the mechanochromic properties exhibited by these terpolymers upon abrasion, with the hybrid architecture of terpolymer **TP5** again showing the most intense response. We estimate the yield of ring-opening reaction in the **TP5** film treated at 500 MPa to be 40% of that of pristine **HO-Rh-OH** treated at this pressure (Figure S20).

To test the reversibility of the color change, the pressure-treated samples **TP3** and **TP5** were heated at 100 °C (Figure S21). At this temperature, the cholesteric phase cleared, and isotropization was achieved. The samples were cooled below an isotropization temperature, and then compression-molded, and cholesteric colors were again observed for both **TP3** and **TP5**, indicating the reversibility of the pressure- and temperature-induced changes in their cholesteric colors.

We previously published a paper on the synthesis, structure, and mechano/thermo/photochromic behaviors of polynorbornene-based homopolymer-functionalized rhodamines [32]. Structural variations in these homopolymers were introduced through (1) the presence of spacer(s) between rhodamine and norbornene and (2) two norbornenes attached at either end of HO-Rh-OH. These two structural elements of the monomer manifest as polymers containing rhodamine with or without spacers between rhodamine and norbornene, as well as non-crosslinked or crosslinked/network systems. Multi-stimuli-responsive properties were observed in the homopolymers composed of spacers on either side of the rhodamine unit, as well as two norbornenes attached at each end of the spacer; this was in sharp contrast to pristine rhodamine, HO-Rh-OH. Not covalently linked to polynorbornene backbone, the latter was unable to undergo reversible ring-opening and closure with the application of stimuli. 

In contrast, the current study deals with terpolymers composed of a liquid crystalline norbornene monomer, a PEG conjugated to norbornene, and rhodamine conjugated to norbornene. We also introduced structural variations: (1) the presence of zero, one, or two spacers between rhodamine and norbornene and (2) the presence of one or two norbornene units on either ends of the rhodamine molecules. These terpolymers self-assembled into various liquid crystalline mesophases within which the dye molecule was enchained. The corresponding LC mesophase–dye interactions and stimuli-responsive properties were analyzed. **TP5** was found to have the best thermochromic, mechanochromic, and piezochromic behaviors among the five terpolymers studied due to the thermochromic cholesteric mesophase and mechanochromic rhodamine molecules undergoing cooperative interaction within a network structure. The mechanochromic behavior of the previously published homopolymer system was observed mostly at the edge of the films [32], while the mechanochromic behaviors of these terpolymers were observed throughout the film. This may be due to the liquid crystalline transitions that are present at and above the glass transition temperature of the terpolymers (35–45 °C).

## 3. Materials and Methods

### 3.1. Instruments and Materials

The instruments and materials used are described in detail in the ESI.

### 3.2. Monomer Syntheses

#### 3.2.1. Monomers 1, 2, 3 and 4

**Monomers 1** through **4** were synthesized as described in the literature [32].

#### 3.2.2. Monomer 5

**Monomer 3** (500 mg, 0.68 mmol), HOBT (138 mg, 1.02 mmol), EDC (209 mg, 1.09 mmol), and 5-norbornene-2-carboxylic acid (103 mg, 0.75 mmol) were dissolved in DMF (10–12 mL) in a round-bottom flask equipped with a stir bar. The reaction mixture was cooled to 0 °C on an ice bath; triethylamine (5–6 mL) was added dropwise to the chilled solution under continuous stirring, whereupon the reaction mixture turned turbid. After completion of the addition of base, the reaction mixture was allowed to gradually warm to room temperature, and the mixture was stirred at ambient T for 12 h. After this time, some precipitation was observed. The reaction mixture was diluted with distilled water, transferred to a separatory funnel, and extracted using CH_2_Cl_2_ (3 × ~10 mL). The combined organic phases were repeatedly washed with brine and dried over anhydrous sodium sulfate (Na_2_SO_4_ anhyd). The volume of the dried solution was reduced by rotary evaporation, and the crude product was loaded onto a flash column (silica; column was packed with hexane mixture of isomers containing triethylamine (3–5 mL). Monitored by TLC, the colorless main product **Monomer 5** was eluted by gradually increasing the polarity of the eluent up to 2:3 ethyl acetate/hexanes. After the removal of the solvent using rotary evaporation, **Monomer 5** (NB-(CH_2_)_10_-Rh-NB) was obtained as a faint yellow, viscous, oily product in 50% yield (290 mg). HR-MS (ESI+, CH_2_Cl_2_): *m*/*z* calculated for C_53_H_63_N_2_O_8_^+^ [M+H]^+^: 855.7214, found 855.7209. A reproduction of the ^1^H NMR spectrum of **Monomer 5** is shown in Figure S6.

### 3.3. Terpolymer Syntheses

Detailed procedures for the syntheses and spectroscopic analyses (NMR spectroscopy, TGA, DSC thermograms, SAXS, and TSAXS data) of the terpolymers **TP1** through **TP5** are presented in the ESI.

## 4. Conclusions

Five different terpolymers containing a norbornene-based cholesteryl LC unit, a norbornene-based PEG unit, and norbornene functionalized with rhodamine were prepared using ROMP. The architectures of the linear or mildly crosslinked terpolymers varied through variations in the rhodamine monomer containing one or two norbornyl moieties that are covalently attached through short or long flexible linkers. The hierarchical self-assembly of the terpolymers films was investigated. In this temperature-controlled morphological study, the co-existence of mesophase and micro-structured domains was shown. The cholesteric mesophase was vitrified and entrapped in the polymer films by processing at the cholesteric mesophase temperature. The corresponding LC mesophase–dye interactions and stimuli-responsive properties were analyzed.

In terpolymers **TP1** and **TP2**, smectic mesophases from the cholesteryl molecules formed the major phases and co-existed with minor amounts of PEG microphase segregated domains; smectic LC–rhodamine interactions were dominant and did not result in cholesteric colors. In **TP3**, smectic mesophases were observed at room temperature along with PEG domains, but they also evolved into a cholesteric mesophase with some smectic enchainment and PEG domains at higher temperatures. **TP4** showed only a minor cholesteric mesophase with dominant smectic mesophases. **TP5** evolved into a cholesteric mesophase with some smectic enchainment and PEG domains at higher temperatures.

The linear polymers **TP1** and **TP3** showed much less favorable mechanochromic properties compared to those of the mildly crosslinked polymers **TP2** and **TP4**. **TP5** combined the flexible linkers of **TP2** and **TP4,** and the mildly crosslinked architectures of **TP2** and **TP4** possessed the greatest mechanochromic color changes among all the five terpolymers. In **TP3** and **TP5**, motional decoupling by the introduction of methylene spacer between the polynorbornene backbone and rhodamine controlled the formation of the cholesteric liquid crystalline phase. **TP5** was found to have the best thermochromic, mechanochromic, and piezochromic behaviors among the five terpolymers due to the thermochromic cholesteric mesophase and the mechanochromic rhodamine molecules undergoing cooperative interaction within a network structure.

**TP5** is characterized by the presence of the unique **Monomer 5,** possessing one long and one short spacer crosslinking it into the terpolymer. The long spacer in this monomer favored the formation of the cholesteric phase, leading to the thermochromic behavior; its shorter spacer enabled mechanochromic behavior. The molecular lengths of **Monomer 5** and the monomer **NBCh9** are similar. This helped in generating a synergistic combination of the mechanochromic property of **Monomer 5** and the thermochromic property of **NBCh9**.

This is the first report of the stimuli-responsive rhodamine dye HO-RH-OH that is incorporated and interacts within LCPs, providing a system that shows both thermochromic and mechanochromic behaviors. **TP5**-like materials are potential candidates for multi-stimuli-responsive materials, where their optical properties can be tuned by mechanical force, thermal energy, and UV light.

## Figures and Tables

**Table 1:** Composition and physical properties of terpolymers **TP1**–**TP5**.

Terpolymer	wt% of Monomers ^a^						wt % Gel Fraction ^b^	M_n_ ^c^, kDa/mol		Đ_M_ ^c^	T_g_ °C ^d^	T_1_ °C ^d^	T_2_ °C ^d^
	NBCh_9_		NBMPEG		NB-Rhodamine Monomer			Theo	GPC				
	Feed	NMR	Feed	NMR	Feed	NMR							
**TP1**	74.9	74.5	15.1	15.3	**9.9**	11.2	-	20.5	16.5	1.2	43.6	82.3	-
					(**Monomer 1**)								
**TP2**	75.3	51.2	14.8	18.6	9.9	30.2	10.2	20.2	-	-	45.0	79.1	-
					(**Monomer 2**)								
**TP3**	74.9	78.1	15.0	13.8	10.1	8.8	-	23.3	19.9	1.3	34.3	67.6	89.4
					(**Monomer 3**)								
**TP4**	75.0	53.4	15.0	19.7	10.0	26.9	14.3	20.0	-	-	39.9	60.4	74.2
					(**Monomer 4**)								
**TP5**	74.6	62.1	15.2	16.6	10.2	21.3	12.6	20.4	-	-	38.6	65.4	87.9
					(**Monomer 5**)								

^a^ Weight% of each monomer in terpolymer is given by feed vs. composition, as determined by ^1^H NMR (400 MHz, CDCl_3_) spectroscopy for **TP1** and **TP3** and ^1^H NMR (400 MHz, HFIP) spectroscopy for lightly crosslinked, solvent swollen terpolymers **TP2**, **TP4**, and **TP5**. Reproductions of ^1^H NMR spectra and further details to calculations are provided in Supporting Information. ^b^ Gel fraction of crosslinked terpolymers were determined by taking weight difference before and after immersion in THF for 7 days. Details of calculations are provided in Supporting Information. ^c^ Mn (number average molecular weight) and ĐM (polydispersity index) were determined by GPC with THF as eluent and calibrated using polystyrene standards. As partially crosslinked polymers, **TP2** and **TP4** were partly insoluble in most of solvents, and thus M_n_ and Ð_M_ were not determined (N/D). ^d^ Thermal transition, T_g_ (glass transition temperature), **T1** and **T2** (LC mesophase transitions), were determined using DSC.

## Data Availability

The original contributions presented in this study are all included in this article/Supplementary Material.

## Supplementary Materials

The following supporting information can be downloaded at: https://www.mdpi.com/article/10.3390/molecules30020401/s1: Figures S1–S6: ^1^H NMR spectra of **Monomer 5** and terpolymers **TP1**–**TP5**; Figure S7: GPC traces of terpolymers **TP1** and **TP3**; Figure S8: TGA traces of terpolymers **TP1**–**TP5;** Figure S9: First cooling DSC traces of terpolymers **TP1**–**TP5**; Figures S10–S14: Room temperature and temperature-dependent small angle X-ray analyses of terpolymers **TP1**–**TP5**; Figure S15: polarized optical micrographs of terpolymers **TP1**–**TP5** at different temperatures; Figure S16–S23: mechanochromic properties of terpolymers **TP1**–**TP5**; Figure S24: UV-visible studies of terpolymers **TP1**–**TP5**.
