# Supplementary material for: Hierarchically Structured Stimuli-Responsive Liquid Crystalline Terpolymer–Rhodamine Dye Conjugates
Source: Molecules. 2025 Jan 18;30(2):401. doi: 10.3390/molecules30020401 (PMC11767871; doi:10.3390/molecules30020401)
Supplement: Supplementary file 1 [file molecules-30-00401-s001.zip › molecules-3397481-supplementary.pdf]

Supporting Information

Hierarchically Structured Stimuli-Responsive Liquid Crystalline Terpolymer–Rhodamine Dye Conjugates

Samiksha Vaidya [1], Meenakshi Sharma [1], Christian Brückner [1,*] and Rajeswari M. Kasi [1,2,*]

[1] Department of Chemistry, University of Connecticut, Storrs, CT 06269, USA
[2] Polymer Program, Institute of Material Science, University of Connecticut, Storrs, CT 06269, USA
* Correspondence: c.bruckner@uconn.edu (C.B.); rajeswari.kasi@uconn.edu (R.M.K.)

ESI Table of Contents

Section		*Page*
Experimental section		S3
i)	Materials	S3
ii)	Synthesis of Monomers and Terpolymers	S4-S6
Characterizations		S7
i)	^{1}H NMR, Mass Spec, GPC, IR, TGA, DSC, UV-vis absorbance	S7
ii)	Morphological properties: SAXS and TSAXS	S8
iii)	Thermochromic properties: UV-vis reflectance, POM	S9
iv)	Mechanochromic and piezochromic properties	S10
Supplementary Figures		S11
i)	^{1}H NMR of **Monomer 5**	S11
ii)	^{1}H NMR of **TP1**, **TP2**, **TP3**, **TP4** and **TP5**	S12-S18
iii)	GPC of **TP1** and **TP3**	S19
iv)	TGA of **TP1**, **TP2**, **TP3**, **TP4** and **TP5**	S20
v)	DSC of **TP1**, **TP2**, **TP3**, **TP4** and **TP5**	S21
vi)	1D SAXS of **TP1**, **TP2**, **TP3**, **TP4** and **TP5**	S22
vii)	T-SAXS of **TP1**, **TP2, TP3 and TP4**	S23-S26
viii)	Polarized optical microscopy of **TP1-TP5**	S27
ix)	Mechanochromic studies (abrasion and compression)	S28-S35
x)	UV-vis absorbance of Terpolymers on adding TFA	S36
References ESI		S37

Experimental Section

Materials:

Materials were used as received: 2-(4-diethylamino-2-hydroxybenzoyl)benzoic acid (98%, TCI America), resorcinol (1,3-benzene diol, 99%, Sigma Aldrich), trifluoroacetic acid (TFA, 99%, Sigma Aldrich), ethanolamine (98%, Sigma Aldrich), anhydrous ethanol (99.5%, Sigma Aldrich), 5-norbornene-2-carboxylic acid (NBCOOH, mixture of *endo*- and *exo*-isomers, 98%, Sigma Aldrich), *N*-(3-dimethylaminopropyl)-*N*′-ethylcarbodiimide hydrochloride (EDC, 98%, Proteochem), hydroxybenzotriazole (HOBt, 97%, Sigma Aldrich), 11-bromoundecanoic acid (99%, Sigma Aldrich), potassium carbonate (K_2CO_3, 99%, Sigma Aldrich), anhydrous dimethylformamide (DMF, 99%, Acros Organics), dry methylene chloride (CH_2Cl_2, DCM, 99.8%, Acros Organics), anhydrous tetrahydrofuran (THF, 99.8%, Acros Organics), triethylamine (99.5%, Sigma Aldrich), pyridine (99.8%, Acros Organics), 2nd Generation Grubbs catalyst ((H_2IMes)(pyr)$_2$(Cl)$_2$RuCHPh (mG2), 98%, AK Scientific), ethyl vinyl ether (EVE, 99%, Acros Organics). NBCh$_9$, a side-chain liquid crystalline monomer, and NBMPEG, a side-chain polyethylene glycol monomer, were synthesized based on previously reported synthetic protocols [1, 2]. Rhodamine (**HO-Rh-OH**) was synthesized using a literature protocol [3].

Synthesis of Monomers:

Syntheses of **Monomers 1-4** were carried out based on previously reported publication[4].

Synthesis of **Monomer 5**

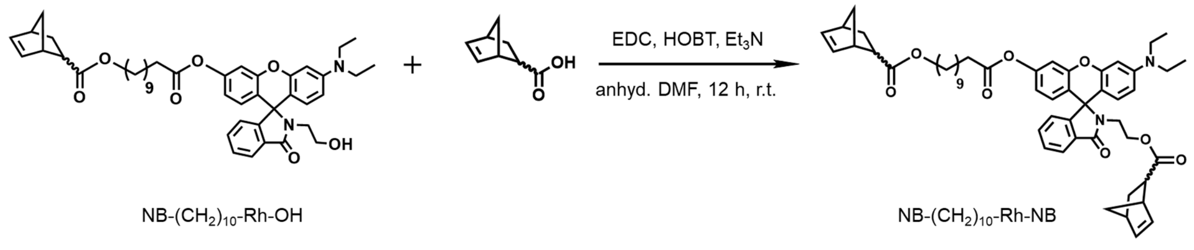

Scheme S1: Synthesis of **Monomer 5**

Monomer 3 (500 mg, 0.68 mmol), HOBT (138 mg, 1.02 mmol), EDC (208 mg, 1.09 mmol), and 5-norbornene-2-carboxylic acid (103 mg, 0.75 mmol) were dissolved in DMF (10–12 mL) in a round-bottom flask equipped with a stir bar. The reaction mixture was cooled to 0 °C on an ice bath; triethylamine (5–6 mL) was added dropwise to the chilled solution with continuous stirring. After the addition of the base was completed, the reaction mixture was allowed to gradually warm to room temperature, and stirred for 12 h at ambient T. The reaction mixture was diluted with distilled water, transferred to a separatory funnel and the product was extracted using CH_2Cl_2 (3 × ~10 mL). The combined organic phase was then washed with brine to remove residual DMF, dried over anhydrous sodium sulfate (Na_2SO_4), and concentrated by rotary evaporation. The crude product was further purified using column chromatography (silica; column packed with hexanes–a mixture of isomers, containing triethylamine (3–5 mL)); the product was eluted by gradually increasing the polarity of the eluent up to 2:3 ethyl acetate: hexane. The fractions were monitored by TLC; the colorless main band was collected to provide, after removal of the solvent using rotary evaporation, **Monomer 5** (NB-$(CH_2)_{10}$-Rh-NB) as a faint yellow viscous oily product in 50% yield (290 mg).

HR-MS (ESI+, CH_2Cl_2): *m/z* calculated for $C_{53}H_{63}N_2O_8^+$ $[M+H]^+$: 855.7214, found 855.7209. ^1H NMR of **Monomer 5** is shown on page S11.

Synthesis of Terpolymers:

We synthesized 5 different random terpolymers (**TP1-5**) by ring-opening metathesis polymerization (ROMP). Each terpolymer is commonly composed of $NBCh_9$ and NBMPEG comonomers along with dye **Monomer 1, 2, 3 4,** or **5** as the third comonomer. Detailed synthesis is described as follows:

Example of the synthesis of a terpolymer:

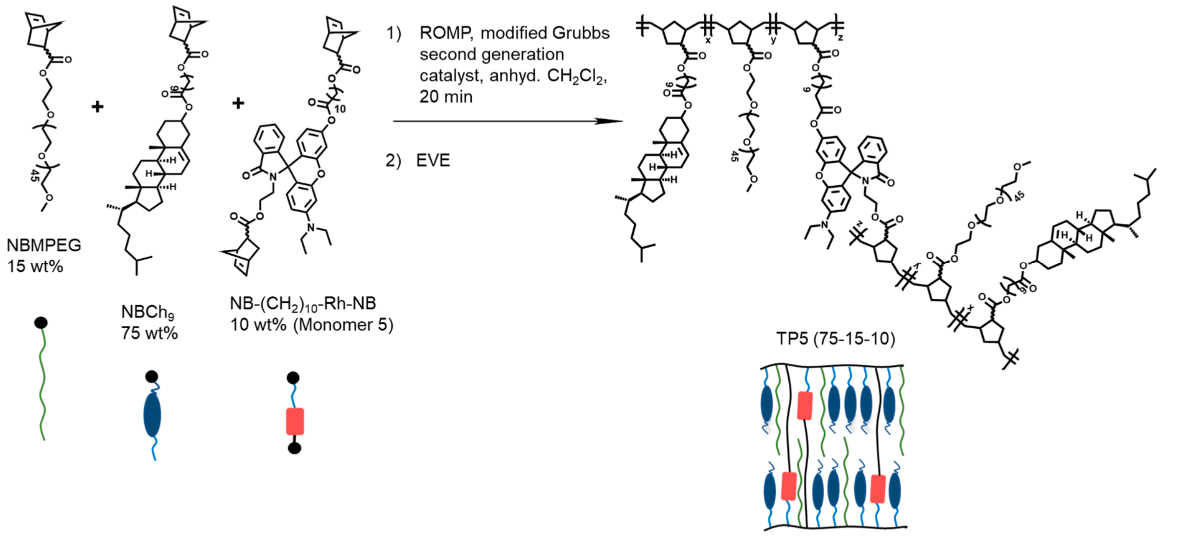

Scheme S2 Representative synthesis of **TP5**

$NBCh_9$ (200 mg, 0.29 mmol), NBMPEG (40.4 mg, 0.02 mmol), and NB-$(CH_2)_{10}$-Rh-NB (Monomer 5, 24.04 mg, 0.03 mmol) was dissolved in CH_2Cl_2 (7 ml) in a 50 ml round bottom flask equipped with a magnetic stir bar and the solution was purged with nitrogen for 5 mins with a needle pierced in the septum for the outlet. In a separate vial, modified Grubbs second generation catalyst (10.66 mg, 0.01 mmol) was dissolved in CH_2Cl_2 (5ml) followed by purging with nitrogen

for 3 mins as described earlier. The Grubbs catalyst solution was then added to the pre-purged monomer solution in the round bottom flask. The reaction was stirred for 15 mins at room temperature and then terminated by adding excess ethyl vinyl ether (EVE). The resulting terpolymer was precipitated in excess methanol, filtered, and dried in a vacuum oven overnight which resulted in a white powder. **TP1**, **TP2**, **TP3,** and **TP4** were synthesized in a similar fashion using **Monomer 1**, **Monomer 2**, **Monomer 3,** and **Monomer 4**, respectively.

^{1}H NMR of **TP1** and **TP3** are shown on page S2 and S3 respectively. In each terpolymer, the amount of NBCh9, NBPEG, and dye monomer enchained were quantified as indicated.

Characterization:

^{1}H NMR spectroscopy was performed on a Bruker DMX 400 MHz NMR spectrometer with DMSO, $CDCl_3$, CD_2Cl_2, or C_4D_8O solvents at room temperature. All the terpolymer samples were analyzed by 1H NMR. Determination of the dye content in the lightly crosslinked solvent swollen gels, for example, TP 2 is more complicated due to the randomness of the chemical conjugation process and this has led to a big difference between dye amounts in the monomer feed and dye conjugated in the terpolymer. In the case of lightly crosslinked terpolymer that is solvent swollen gels, we can provide only an estimation of the comonomer % with the understanding that the error % will be more for crosslinked terpolymers (TP2, TP4, and TP5) than non-crosslinked terpolymers (TP1 and TP3).

Mass spectra were obtained on a Micromass Quattro-II triple quadruple mass spectrometer equipped with an electrospray ionization (ESI) mode source.

Gel permeation chromatography (GPC) was performed using a Waters 1515 instrument, coupled with a PL-ELS1000 evaporative light scattering (ELS) detector and a Waters 2487 dual wavelength absorbance UV-vis detector, using tetrahydrofuran (THF) or dimethylacetamide (DMAc) as eluents, and polystyrene (PS) standards for constructing a conventional calibration curve.

Differential scanning calorimetry (DSC) of all the five terpolymers was performed on a TA-2920 instrument (Q-100 series), calibrated with an indium standard; 5–10 mg sample quantities were tested at scanning rates of 10 °C/min; phase transition temperatures were determined during the first cooling cycle using Universal Analysis software.

FTIR studies of lightly crosslinked terpolymers **TP2**, **TP4,** and **TP5** were carried out on a Nicolet Magna 560 FT/IR Specac Quest–single refection diamond crystal ATR instrument, at 64 scans, with a resolution of 4 cm^{-1}.

UV-vis spectra of linear terpolymer samples **TP1** and **TP3** dissolved in DCM or THF were recorded using 1 x 1 cm quartz cells using a Cary 50 (Varian) instrument before and after the addition of TFA.

Morphological properties: Small Angle X-Ray Scattering (SAXS) and Temperature-controlled SAXS (T-SAXS)

All five terpolymer samples were compression molded based on the thermal transitions observed from their DSC thermograms. Terpolymers **TP1** and **TP2** were compression molded around 75 °C, whereas samples **TP3** and **TP4** were compression molded between 60 °C and 88 °Cto obtain free-standing solid films. SAXS was performed on a pin-hole collimated Rigaku SMAX3000 instrument configured with CuKα radiation (1.542 Å) produced by a micro-focus source; the beam diameter on the sample plane was 1 mm. Scattering intensities were recorded on a gas-wire electronic area (2D) detector with a resolution of 1024 × 1024 pixels, located at a distance of ~80 cm from the sample center, permitting access to scattering vectors ranging from 0.015–0.22 $Å^{-1}$; silver behenate (d-spacing = 58.38 Å) was used to calibrate the SAXS diffraction patterns. 1D representation of the scattered intensity against scattering vector q of TP1-5 was obtained using MATLAB routines (Rigaku) using equation$q = (4\pi/\lambda) \times \sin\theta$ with the scattering angle of 2θ.

For TSAXS, samples sandwiched between the Kapton sheets were subjected to a heating/cooling rate of 5 °C/min and held for 5 min at each temperature prior to 10 min data acquisition within the temperature range of 25-110 °C.

Optical properties:

UV-vis reflectance: Selectively light-reflecting terpolymers and control copolymer blended with **HO-Rh-OH** [P(NBCh$_9$-r-NBMPEG) physically mixed with rhodamine dye **HO-Rh-OH**] were prepared by compression molding and annealing samples (~200 mg) in the temperature range of 70-90 °C between Kapton sheets for about 30 min. Any cholesteric structure present in these materials was locked by quenching the sample to room temperature ($T < T_g$) by using compressed air. Typically, the terpolymer films with approximately ~ 0.2 mm thickness were obtained. UV-vis spectra of these terpolymer films were recorded on a Shimadzu UV-vis spectrometer (UV-2450) in reflectance mode with a wavelength range of 200-800 nm.

Polarized Optical Microscopy: An Olympus BX51P microscope equipped with an Instec HCS410 hot stage was used to determine the optical textures of the films. The samples were placed between two coverslips and annealed between the temperature window from T_{LC1} to T_{LC2} transition temperatures in the case of **TP3** and **TP5** for 24 h, and the characteristic texture of the mesophases was recorded. Samples **TP1**, **TP2,** and **TP4** had only one LC thermal transition temperature and these samples were annealed around that one LC thermal transition.

Mechanochromic and piezochromic properties

The terpolymer samples and control sample [P(NBCh9-r-NBMPEG) blended with **HO-Rh-OH** and placed on a white sheet of paper and abraided with a pestle. Photographic images were captured as the terpolymers gave a visible change in color. Heating the abraided terpolymers at 100 °C led to a reversible change to the original color which was captured again by a camera. The bright and strong mechanochromic color change of **TP5** to dark red was also observed under UV 365 nm UV light as a red color change.

The powder samples were mixed well and compression molded to produce several samples for testing mechanochromic behaviors. At least three samples of each type before and after mechanical stress were photographed and the average color intensities were established by ImageJ analysis of the photographs. Raw histogram data of some of the samples and the controls are provided in the ESI.

For studying the piezochromic properties the powders of each terpolymer were placed into a pellet pressing die, which was then placed into a Carver press and the terpolymers were subjected to 500 MPa pressure. The terpolymer film thus formed was carefully removed and the color change upon film formation was recorded.

Supplementary Figures

Entry	Dye monomer	Chemical Structure	Schematic
TP1	Monomer 1 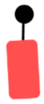	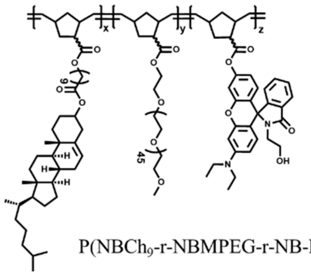 P(NBCh$_9$-r-NBMPEG-r-NB-Rh-OH)	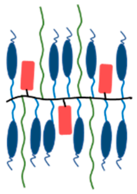
TP2	Monomer 2 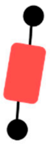	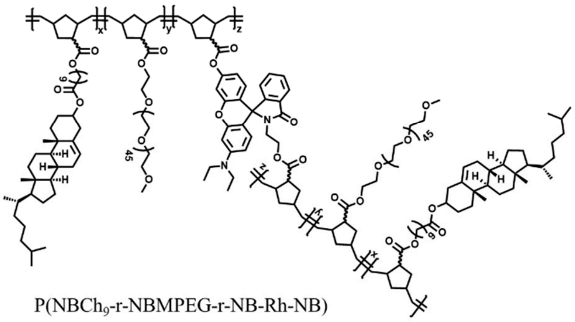 P(NBCh$_9$-r-NBMPEG-r-NB-Rh-NB)	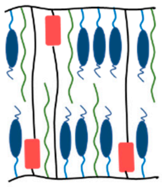

Table S1. Structures and schematic representations of **TP1** and **TP2** derived from the ring-opening metathesis polymerization (ROMP) of Monomer 1 and Monomer 2 respectively along with two co-monomers NBCh9 and NBMPEG by using modified Grubbs second generation catalyst in dichloromethane (CH_2Cl_2) or tetrahydrofuran (THF); termination of the polymerization by addition of ethyl vinyl ether (EVE). **TP1** (using monofunctional **Co-Monomer 1**); **TP2** (using bifunctional without spacer: Co-Monomer 2)

Entry	Dye monomer	Chemical Structure	Schematic
TP3	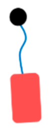 Monomer 3	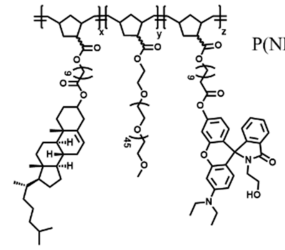 P(NBCh$_9$-r-NBMPEG-r-NB-(CH$_2$)$_{10}$-Rh-OH)	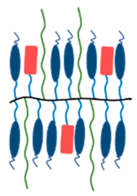
TP4	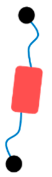 Monomer 4	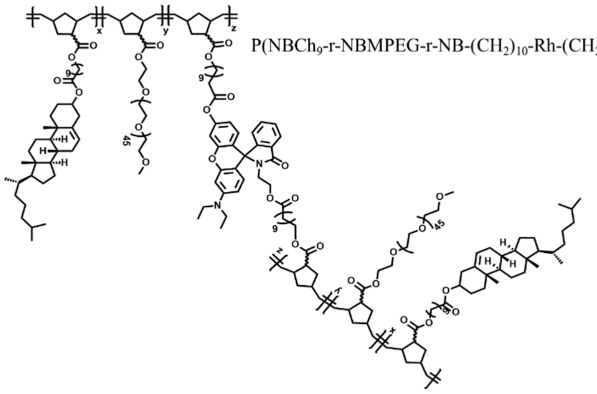 P(NBCh$_9$-r-NBMPEG-r-NB-(CH$_2$)$_{10}$-Rh-(CH$_2$)$_{10}$-NB)	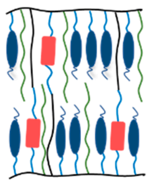
TP5	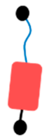 Monomer 5	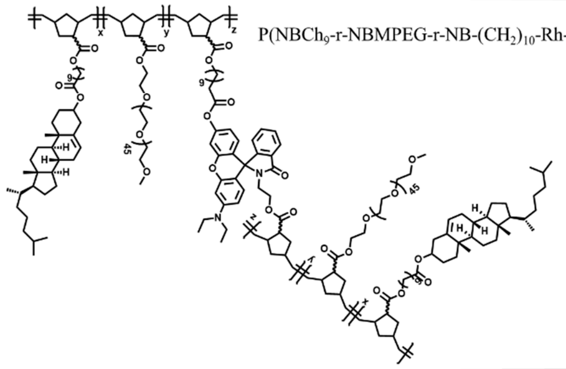 P(NBCh$_9$-r-NBMPEG-r-NB-(CH$_2$)$_{10}$-Rh-NB)	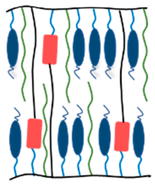

Table S2. Structures and schematic representations of **TP3** through **TP5** derived from the ring-opening metathesis polymerization (ROMP) of **Monomer 3**, **Monomer 4** and **Monomer 5** respectively along with two co-monomers NBCh9 and NBMPEG by using modified Grubbs second generation catalyst in dichloromethane (CH_2Cl_2) or tetrahydrofuran (THF); termination of the polymerization by addition of ethyl vinyl ether (EVE). **TP3** (using monofunctional with spacer on one side: **Co-Monomer 3**); **TP4** (using bifunctional with spacer on both sides: **Co-Monomer 4**), **TP5**: (using bifunctional with spacer only on one side (hemispacer): **Co-Monomer 5**).

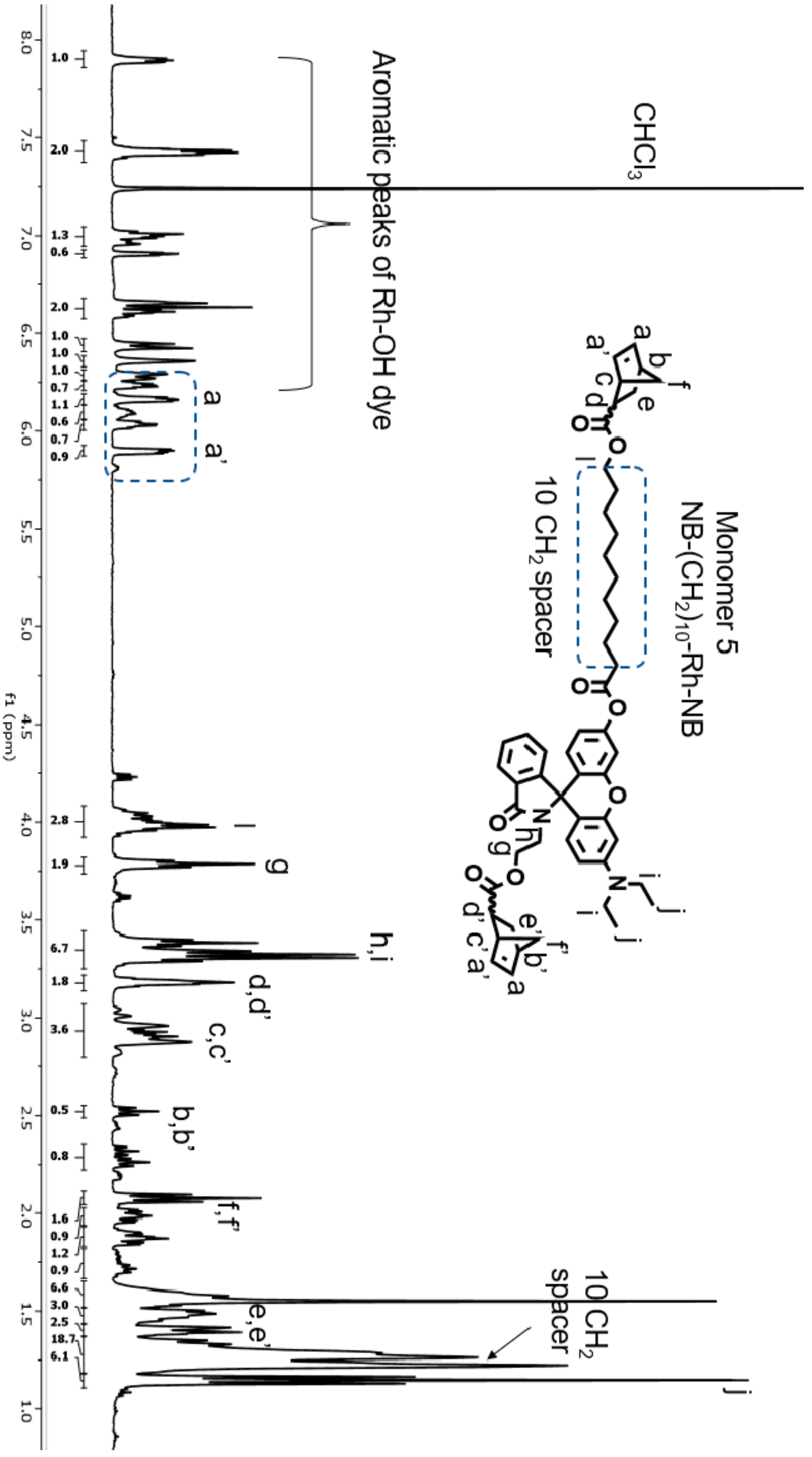

Figure S1 ^{1}H NMR ($CDCl_3$, 400 MHz) spectrum of **Monomer 5** (NB-$(CH_2)_{10}$-Rh-NB) collected at room temperature.

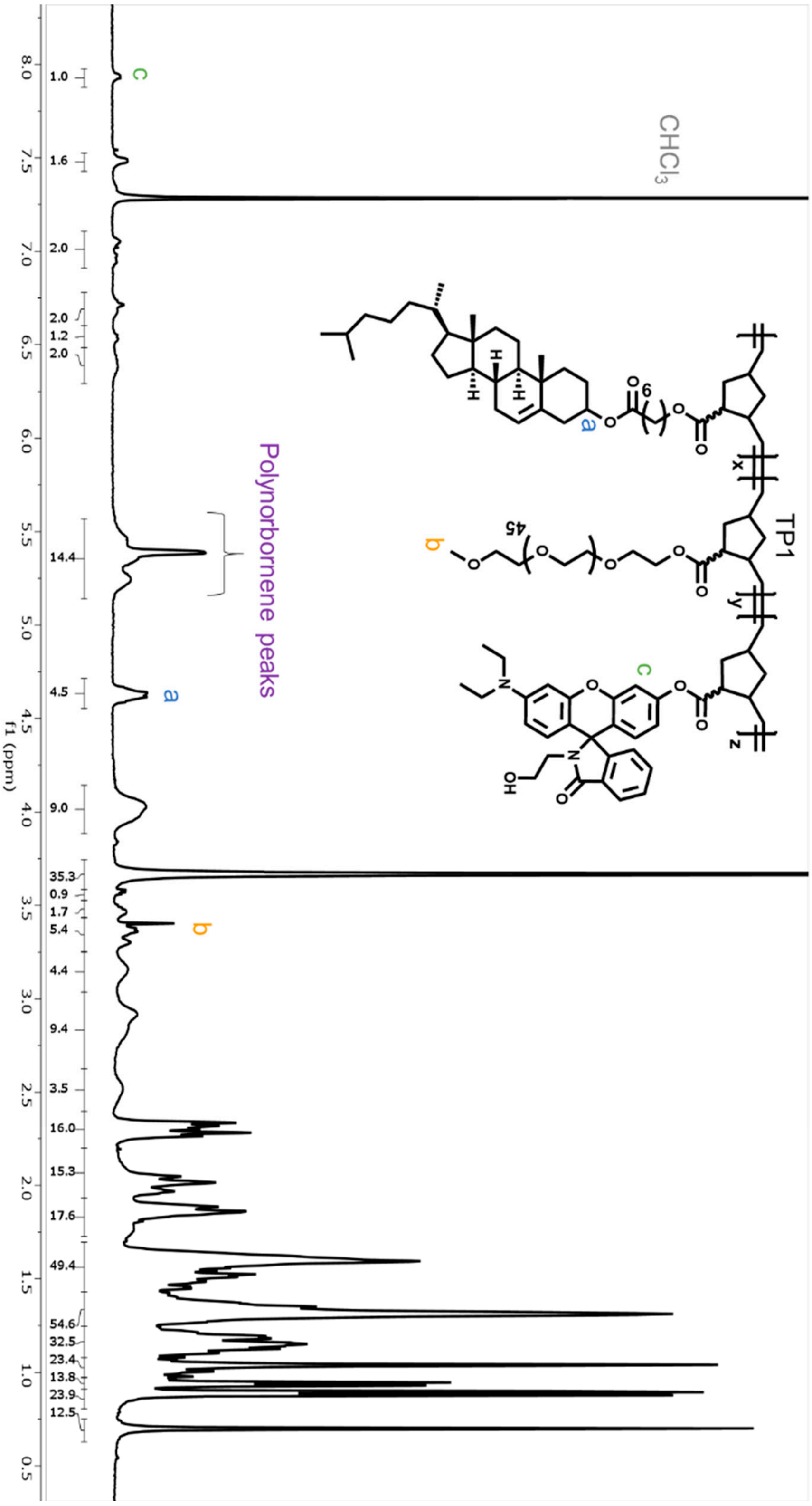

Figure S2. ^{1}H NMR ($CDCl_3$, 400 MHz) spectrum of **TP1** collected at room temperature.

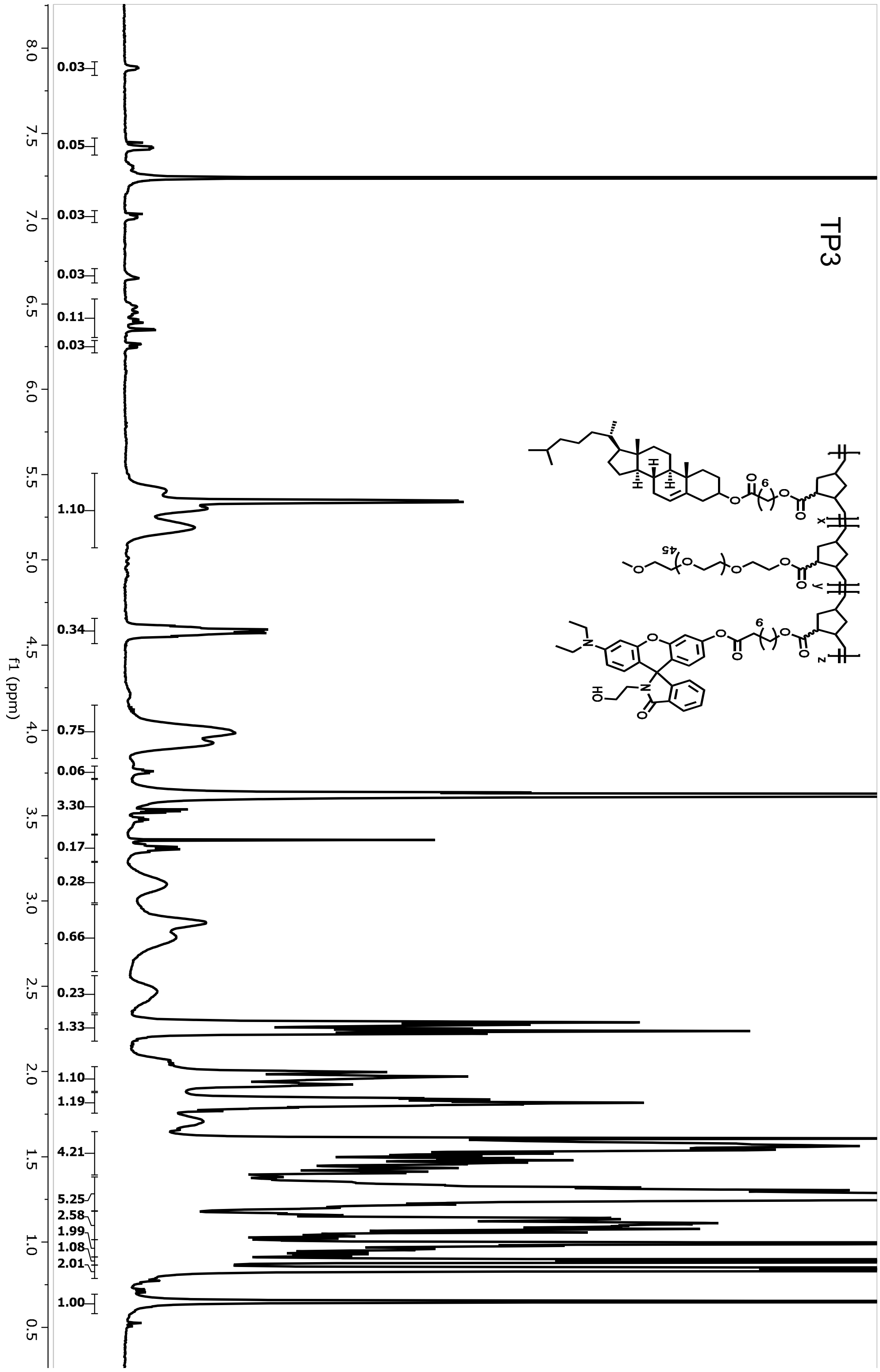

Figure S3. ^{1}H NMR ($CDCl_3$, 400 MHz) spectrum of **TP3** collected at room temperature.

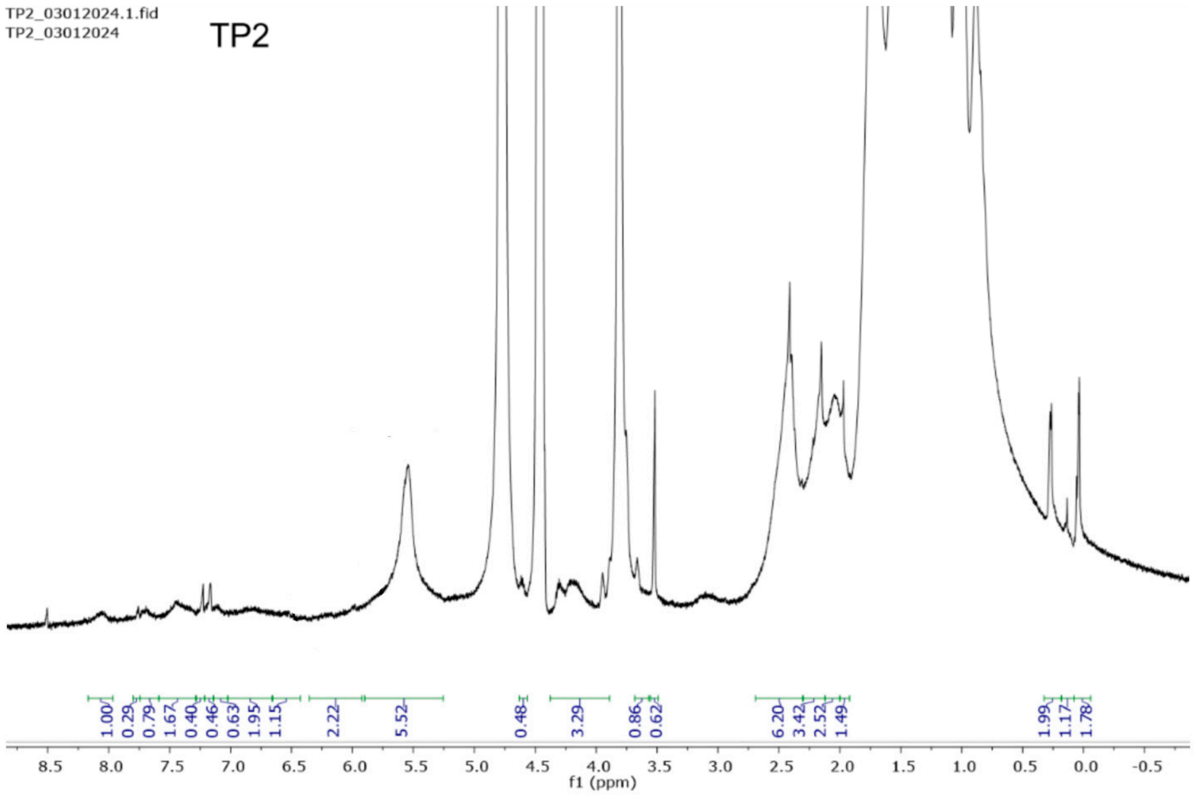

Figure S4. ^{1}H NMR (HFIP, 400 MHz) spectrum of solvent swollen **TP2** collected at room temperature.

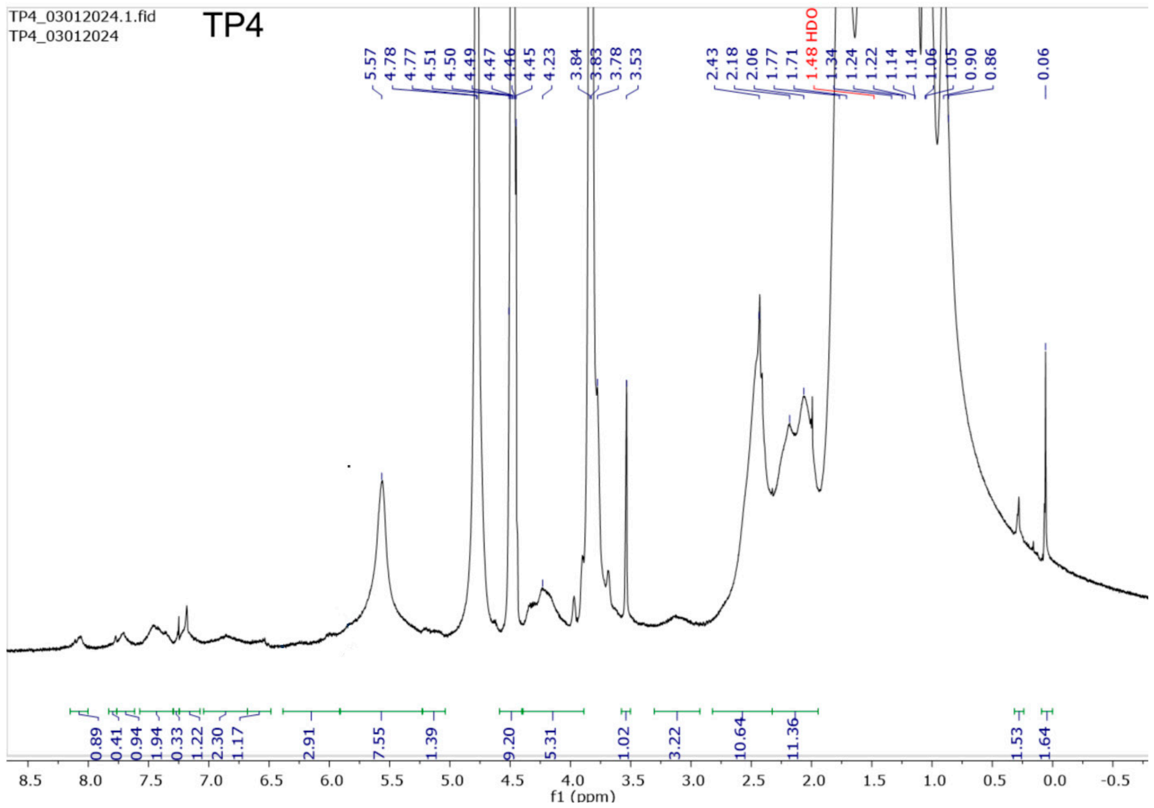

Figure S5. ^{1}H NMR (HFIP, 400 MHz) spectrum of solvent swollen **TP4** collected at room temperature.

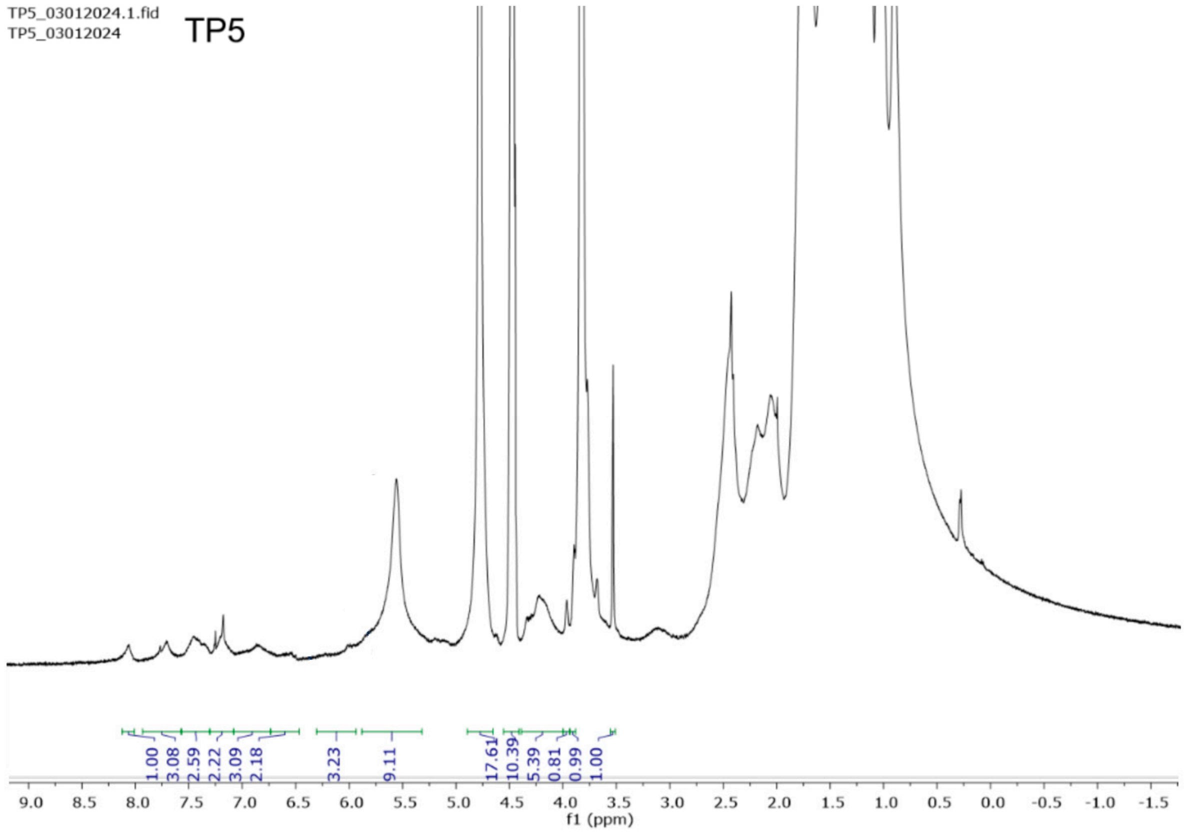

Figure S6. ^{1}H NMR (HFIP, 400 MHz) spectrum of solvent swollen **TP5** collected at room temperature.

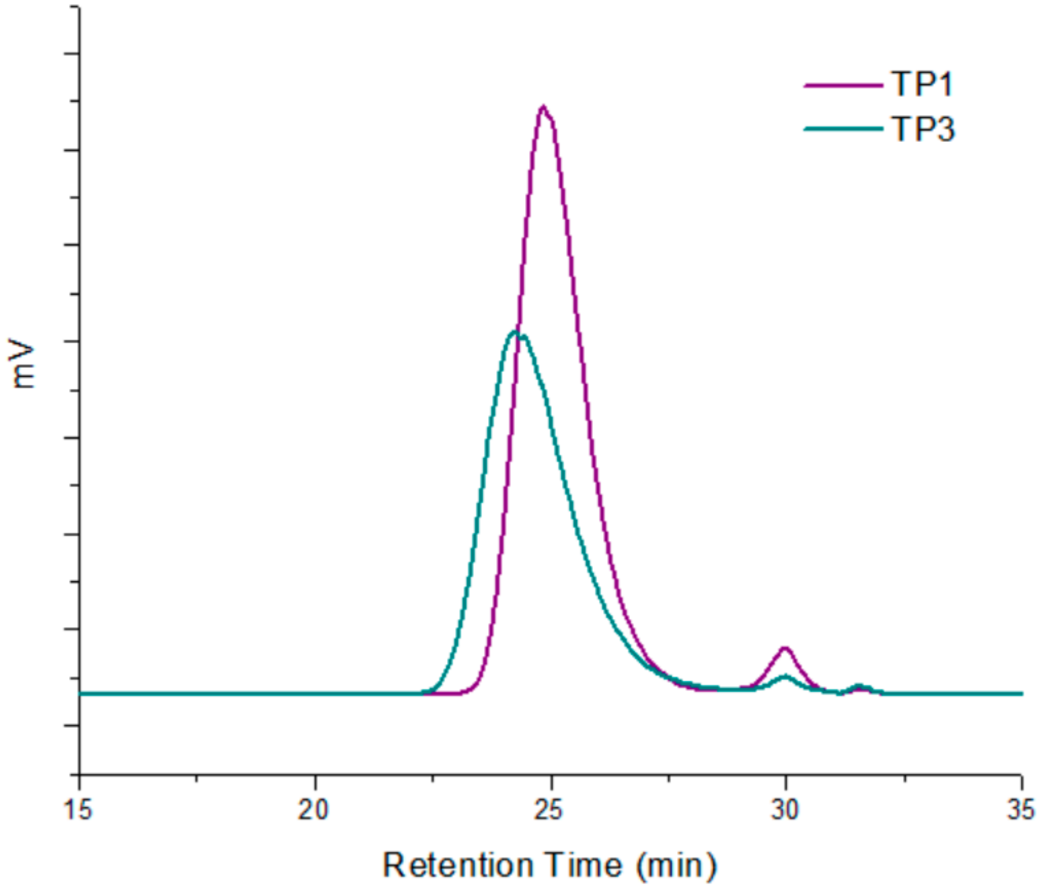

Figure S7. GPC traces of **TP1** and **TP3** at room temperature with THF as the eluent calibrated using PS standards.

TGA of Terpolymers:

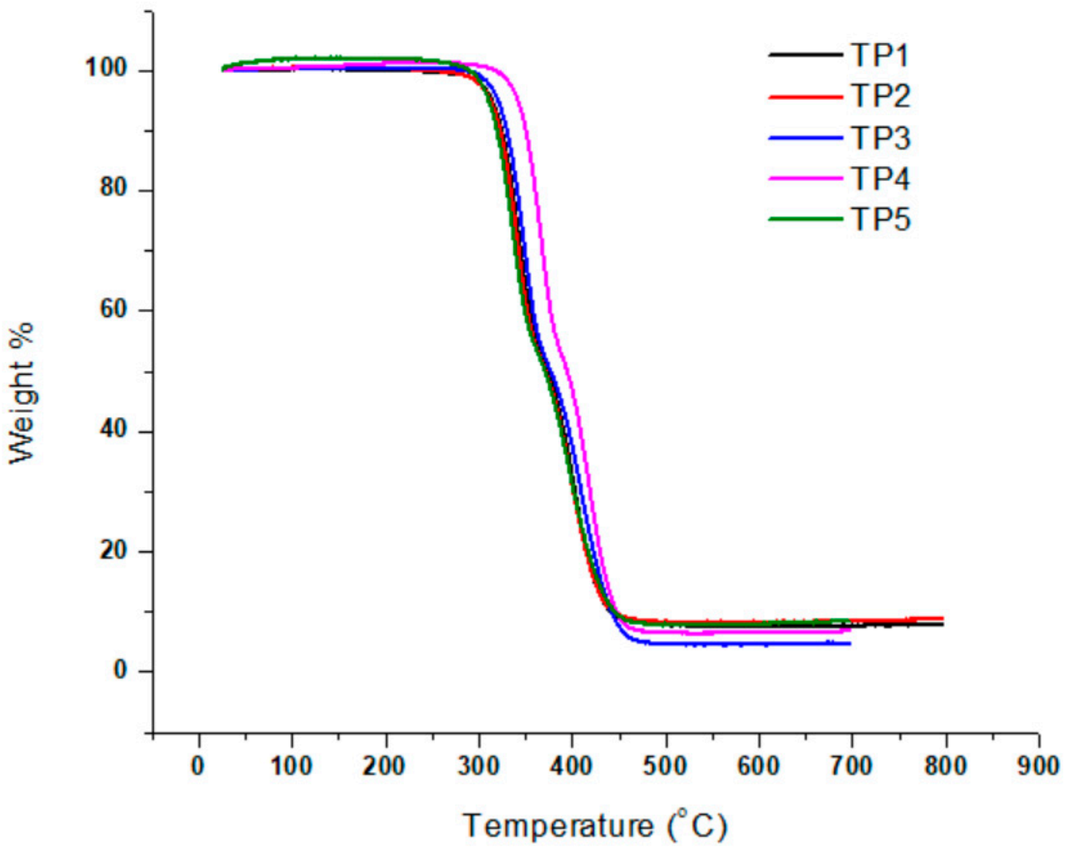

Figure S8. TGA of Terpolymers **TP1** through **TP5**

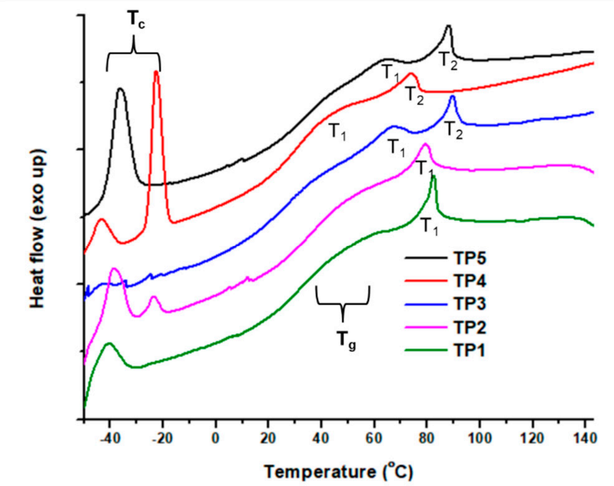

Terpolymers	T_g °C	T_1 °C	T_2 °C
TP1	43.6	82.3	
TP2	45.0	79.1	
TP3	34.3	67.6	89.4
TP4	39.9	60.4	74.2
TP5	38.6	65.4	87.9

Figure S9. First cooling DSC curves of Terpolymers: **TP1** through **TP5** T_c = PEG crystallization temperature, T_g = Glass transition temperature for each polymer T_1 = First thermal transition in each polymer, T_2 = second thermal transition observed in **TP3**, **TP4** and **TP5**

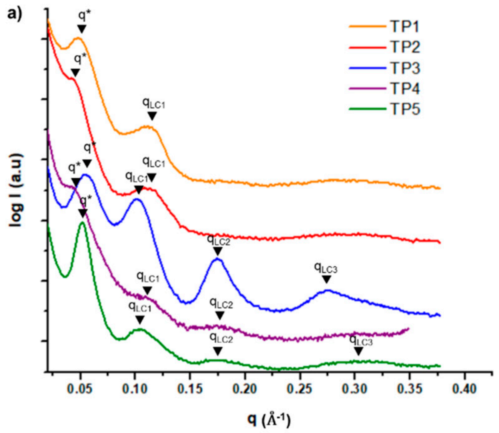

b)

Terpolymer	q*(nm)	d_1	d_2	d_3	Side chain length
TP1	12.56	5.23	-	-	3.34
TP2	15.7	5.23	-	-	3.34
TP3	10.5	6.3	3.6	2.28	3.34
TP4	13.96	5.23	3.5	-	3.34
TP5	11.4	5.98	3.6	2.06	3.34

Figure S10. Room temperature Small Angle X Ray Scattering (SAXS) of Terpolymers **TP1**, **TP2**, **TP3**, **TP4** and **TP5**. a) 1D-SAXS diffractogram where the scattering vector q* represents side chain segregation of PEG, d_1, d_2 and d_3 are assigned to q_{LC1}, q_{LC2} and q_{LC3} which are reflections from LC ordering. b) Table provides the d-spacing values from the diffractogram.

The room temperature SAXS are carried out on each of the five terpolymers **TP1** through **TP5**. The d-spacing values for each of the peaks q*, q_{LC1}, q_{LC2}, q_{LC3} are calculated and compared with the side chain length of the majority monomer (NBCh9). The side chain length shown in the table corresponds to that of NBCh9 molecule as determined by CHEM 3D. q* peak of **TP2** and **TP4** is broad and not very distinct. Also, in case of **TP4**, the peaks are broad so the center of the broad peak is chosen for calculating the d value.

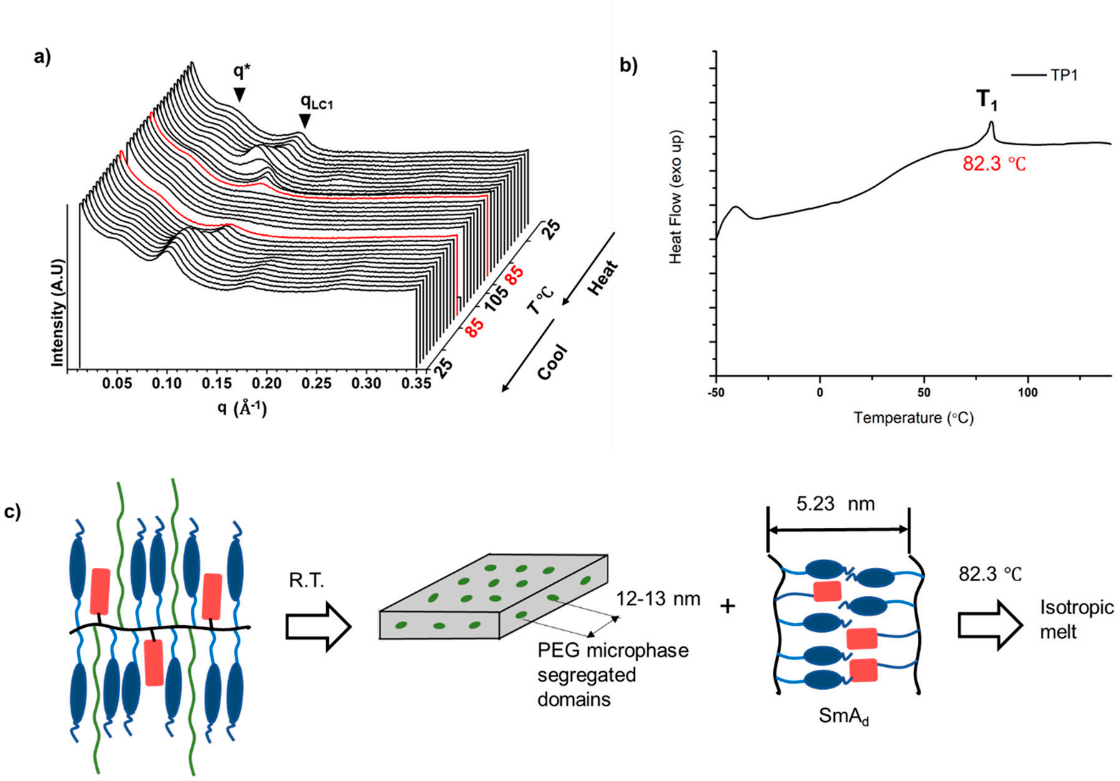

Figure S11. Structure, temperature-dependent SAXS measurement and mesophase evolution for **TP1**: a) 1D data derived from temperature-dependent SAXS. b) First cooling DSC cycle c) Phase evolution of **TP1** during heating cycle is demonstrated.

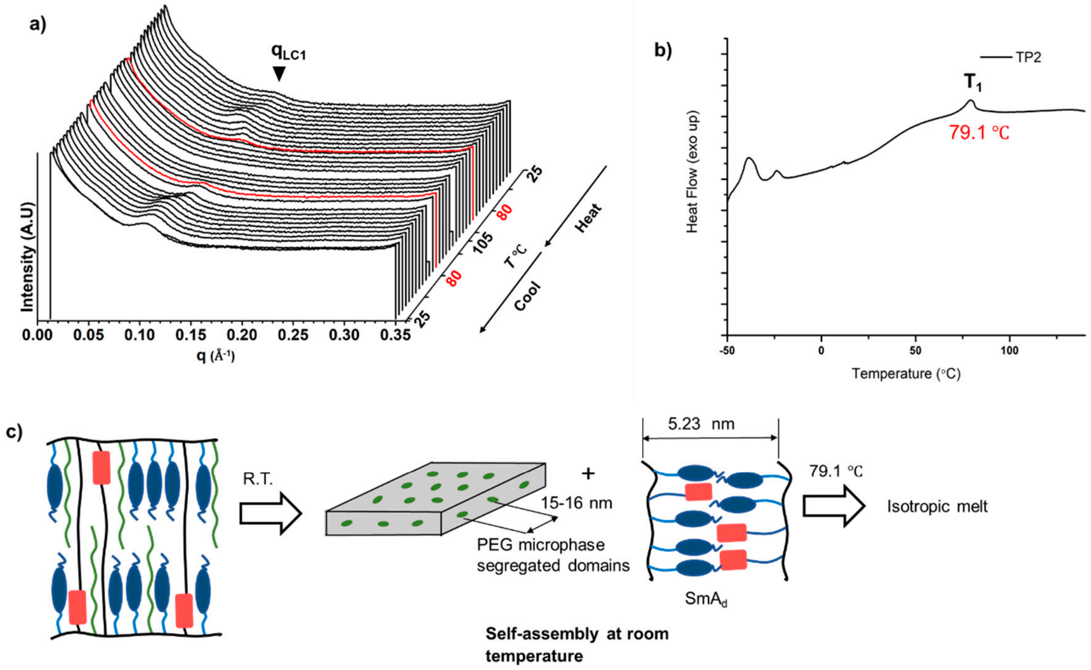

Figure S12. Structure, temperature-dependent SAXS measurement and mesophase evolution for **TP2**: a) 1D data derived from temperature-dependent SAXS b) First cooling DSC cycle c) Phase evolution of **TP2** during heating cycle is demonstrated.

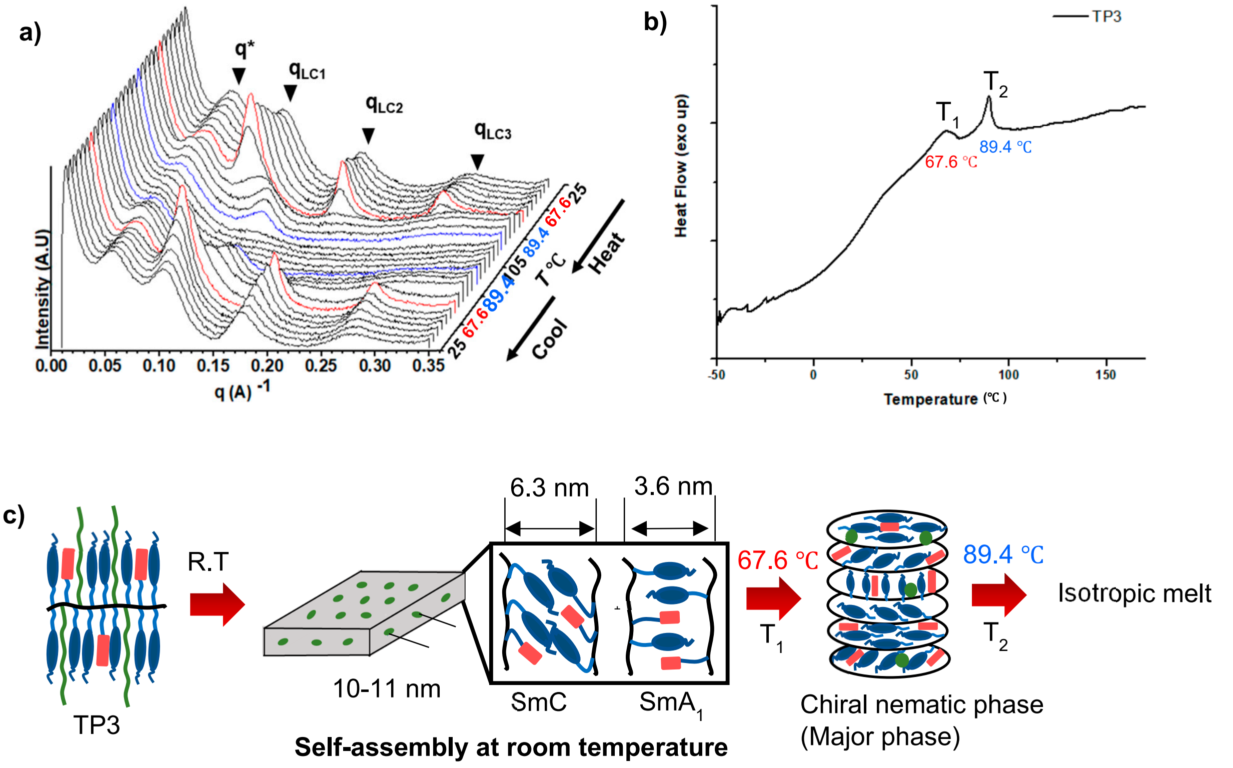

Figure S13. Temperature-dependent SAXS measurement and mesophase evolution for TP3: a) temperature-dependent SAXS 1D data for **TP3**. b) First cooling DSC cycle. c) Graphic representation of the phase evolution of **TP3** derived from the SAXS and DSC data.

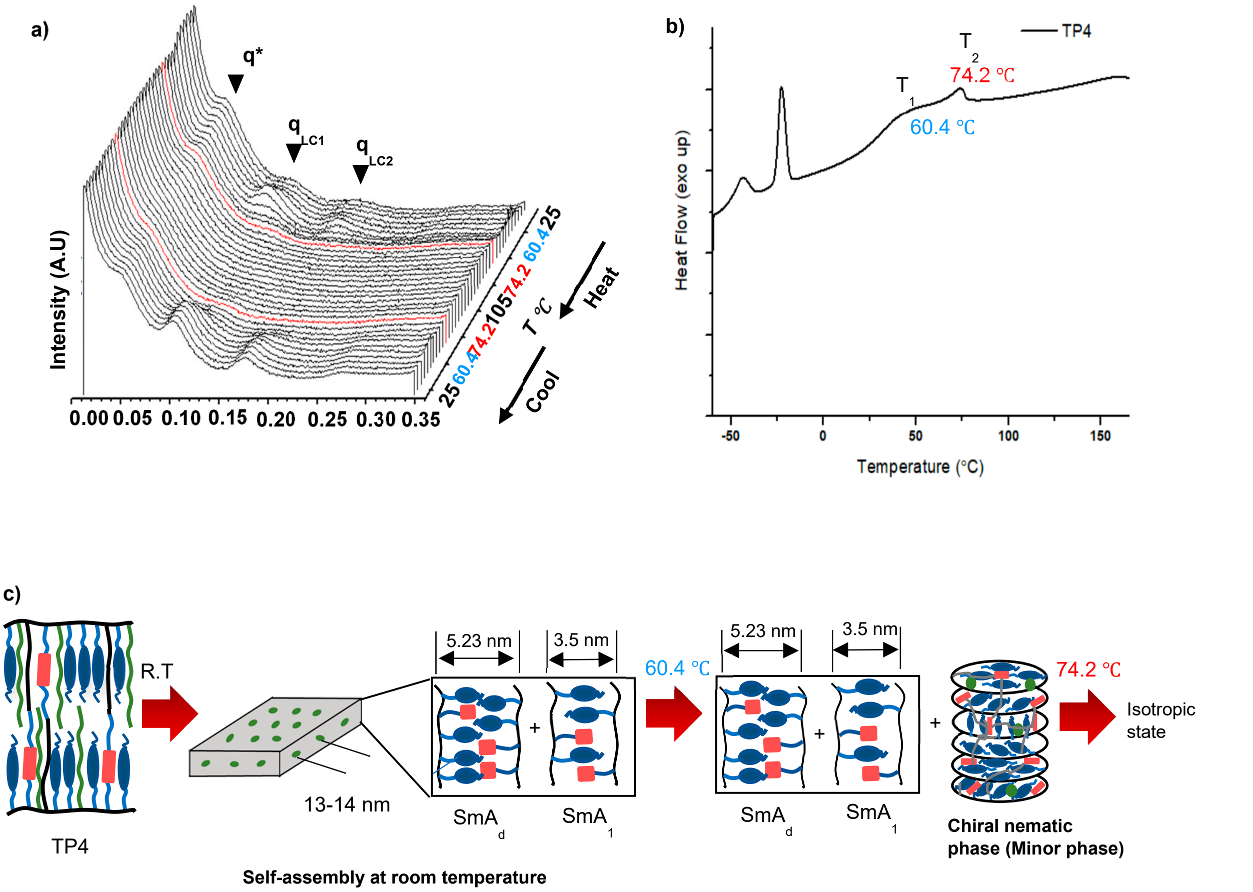

Figure S14. a) 1D data derived from temperature-dependent SAXS of **TP4**, b) First cooling DSC cycle of TP4 and c) Phase evolution of **TP4** during heating cycle is demonstrated.

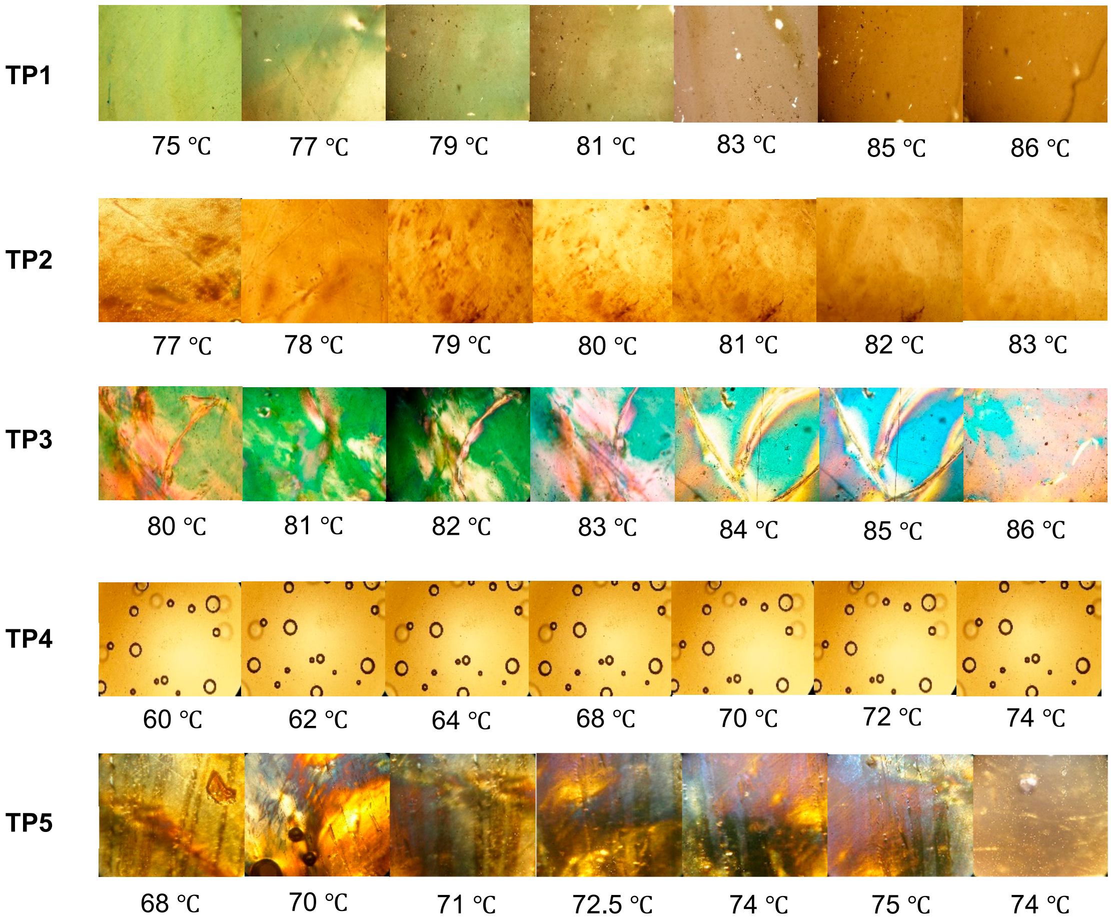

Figure S15. Polarized Optical Microscopy images. Cholesteric fingerprint oily streak observed in **TP3** and **TP5** and absent in **TP1**, **TP2** and **TP4**.

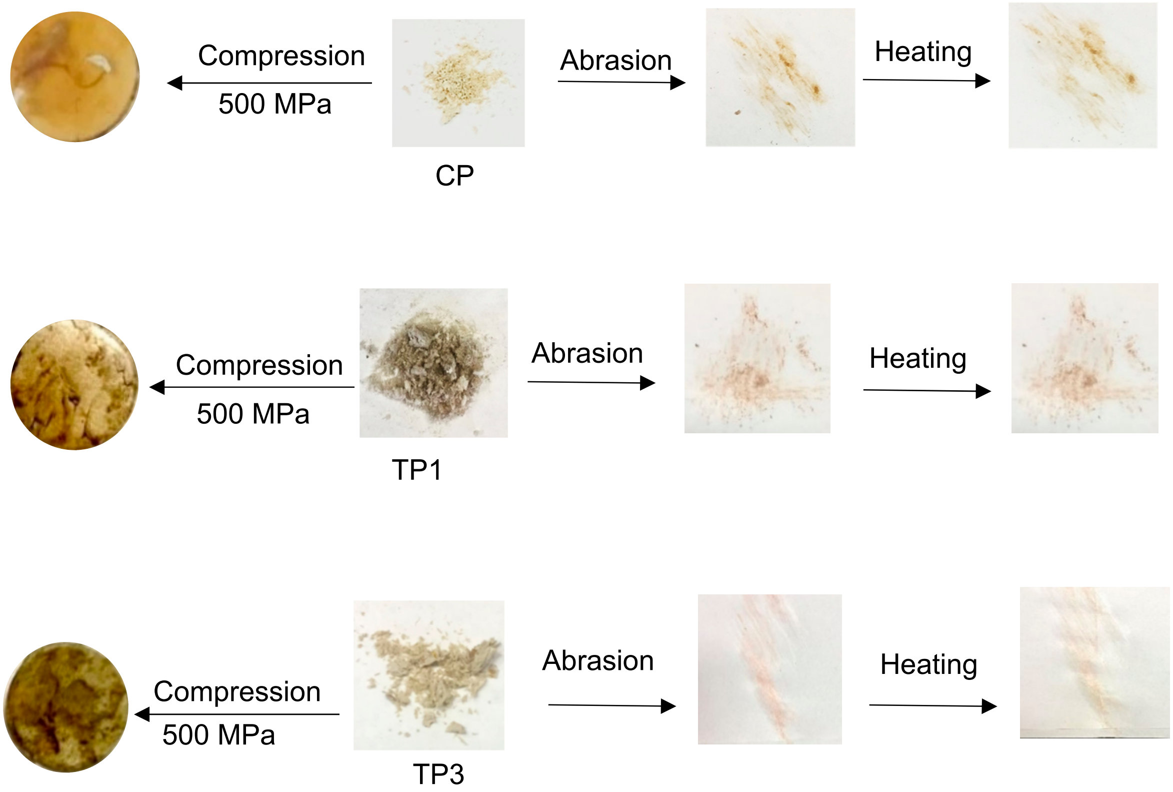

Figure S15. Deficient mechanochromic upon compression (500 M Pa, left) and abrasion (right)of **TP1**, **TP3** and control sample (**CP**).

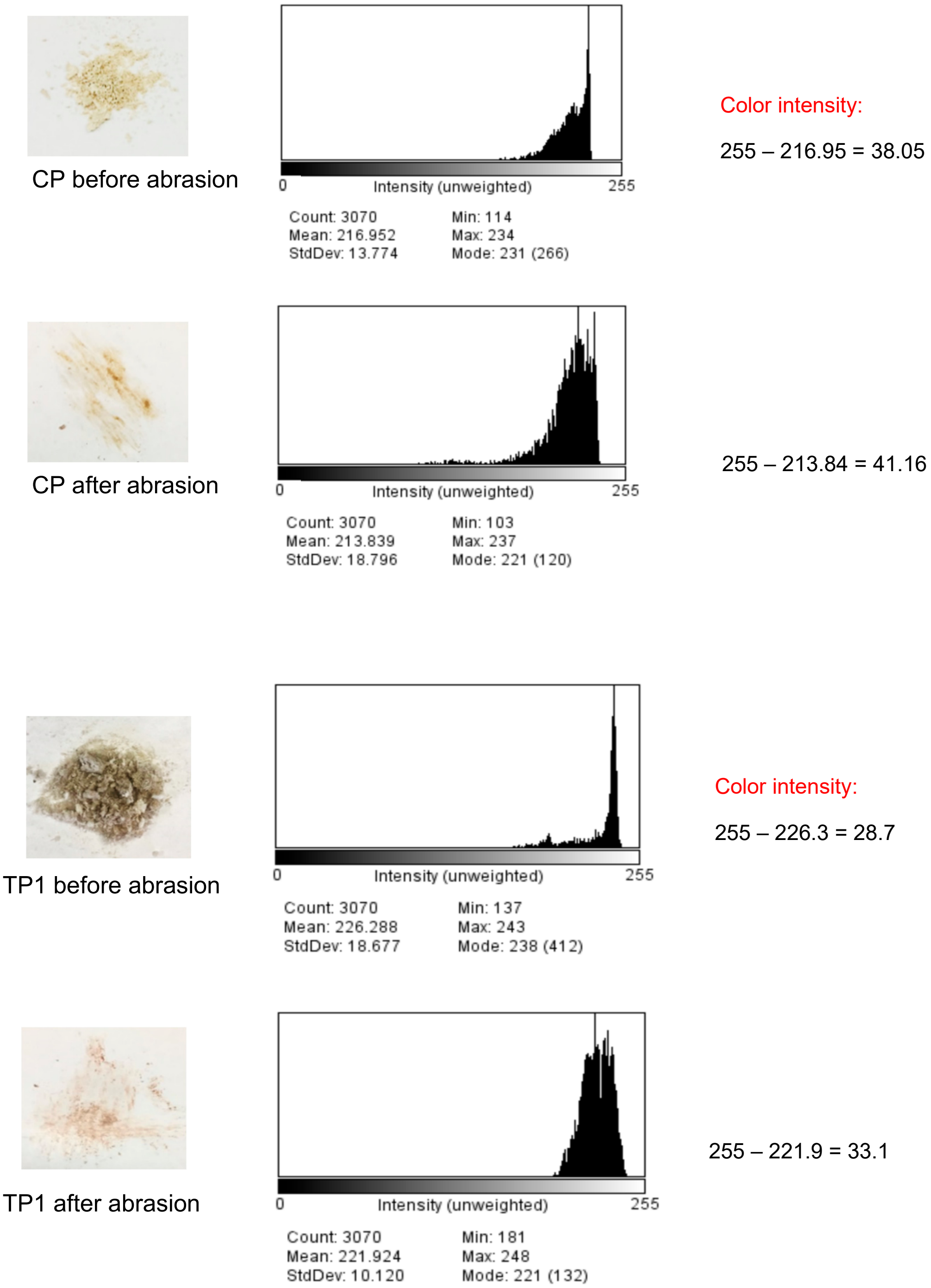

Figure S16. Example method used for quantifying colorimetric intensity of Copolymer (**CP**) and **TP1** before and after abrasion using ImageJ software. The value 255 is considered as pure white according to the gray scale. Both **CP** and **TP1** do not present mechanochromic behavior.

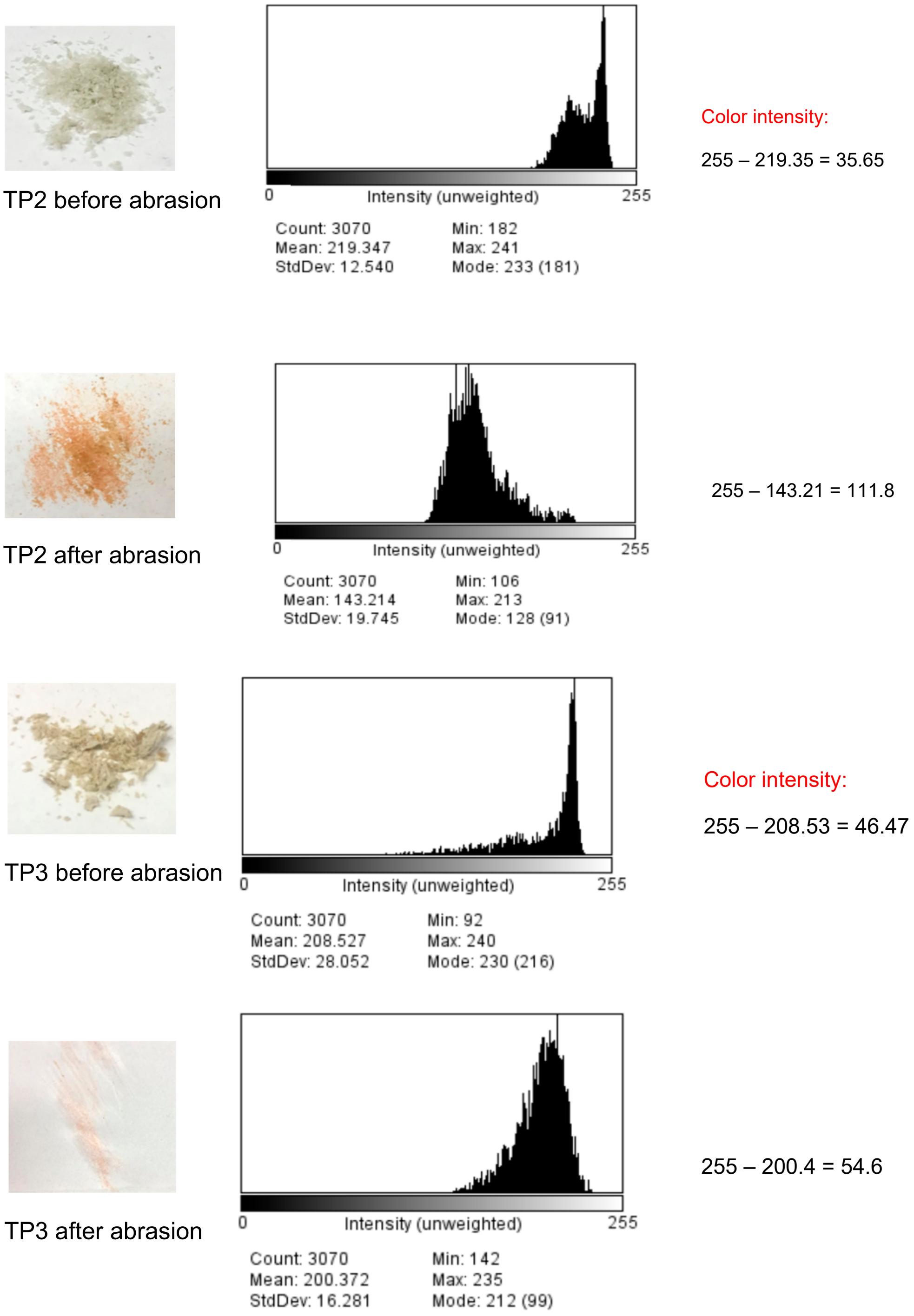

Figure S18. Deficient mechanochromic behavior of **TP2** and **TP3**. Example method used for quantifying colorimetric intensity of **TP2** and **TP3** before and after abrasion using ImageJ software. The value 255 is considered as pure white according to the gray scale.

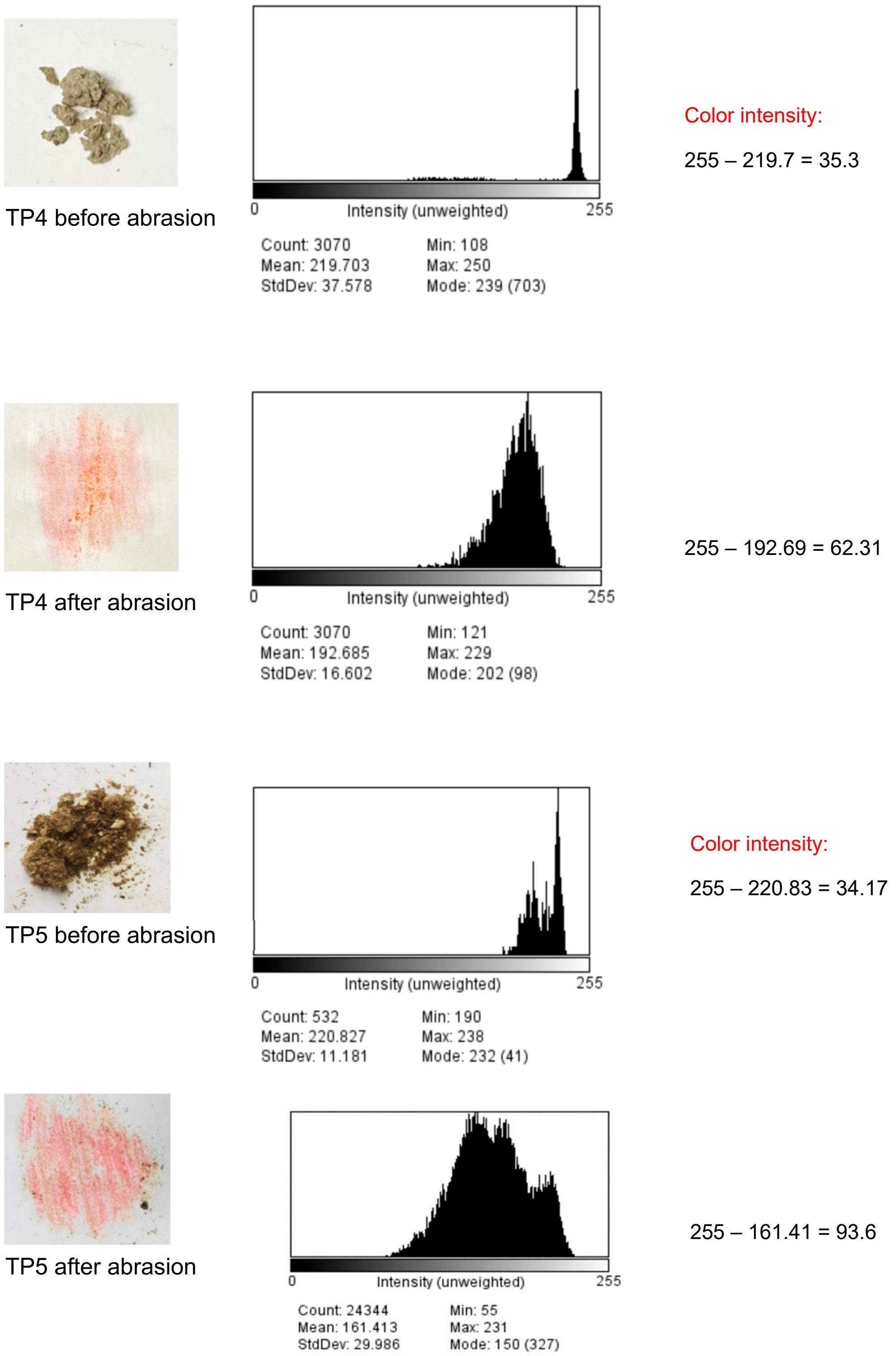

Figure S19. Mechanochromic behavior of **TP4** and **TP5**. Example method used for quantifying colorimetric intensity of **TP4** and **TP5** before and after abrasion using ImageJ software. The value 255 is considered as pure white according to the gray scale.

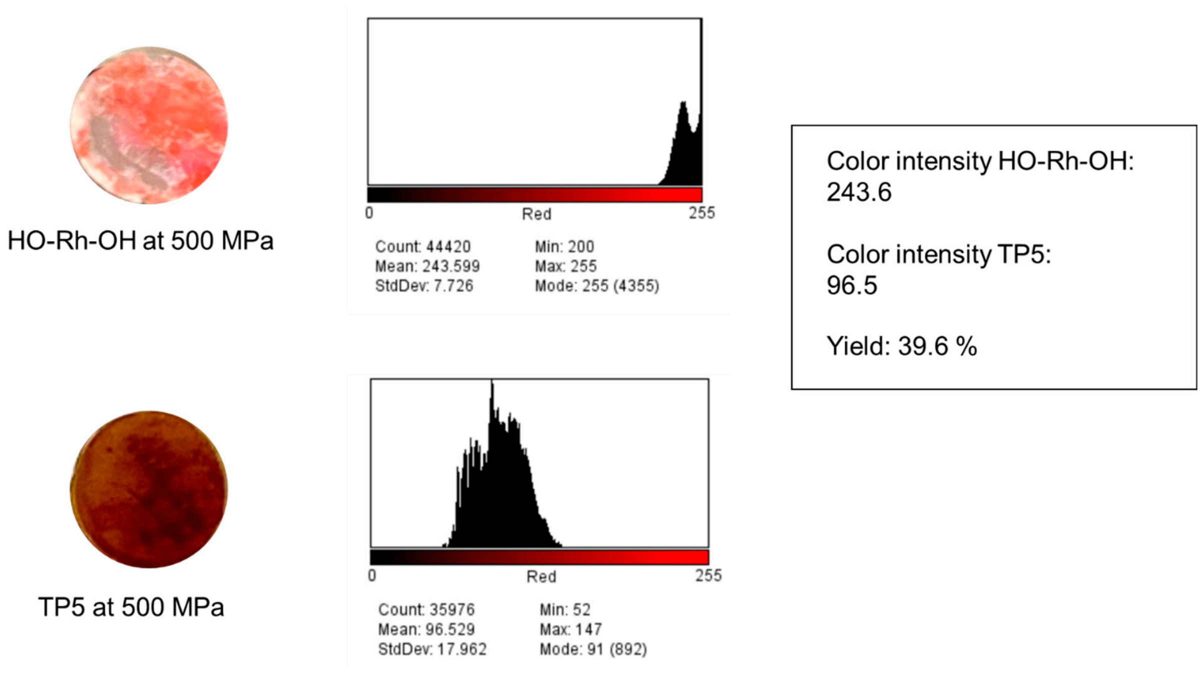

Figure S20. Yield of ring-opening of **HO-Rh-OH** in **TP5**. Comparing intensities of **HO-Rh-OH** and **TP5** after treatment at 500 MPa.

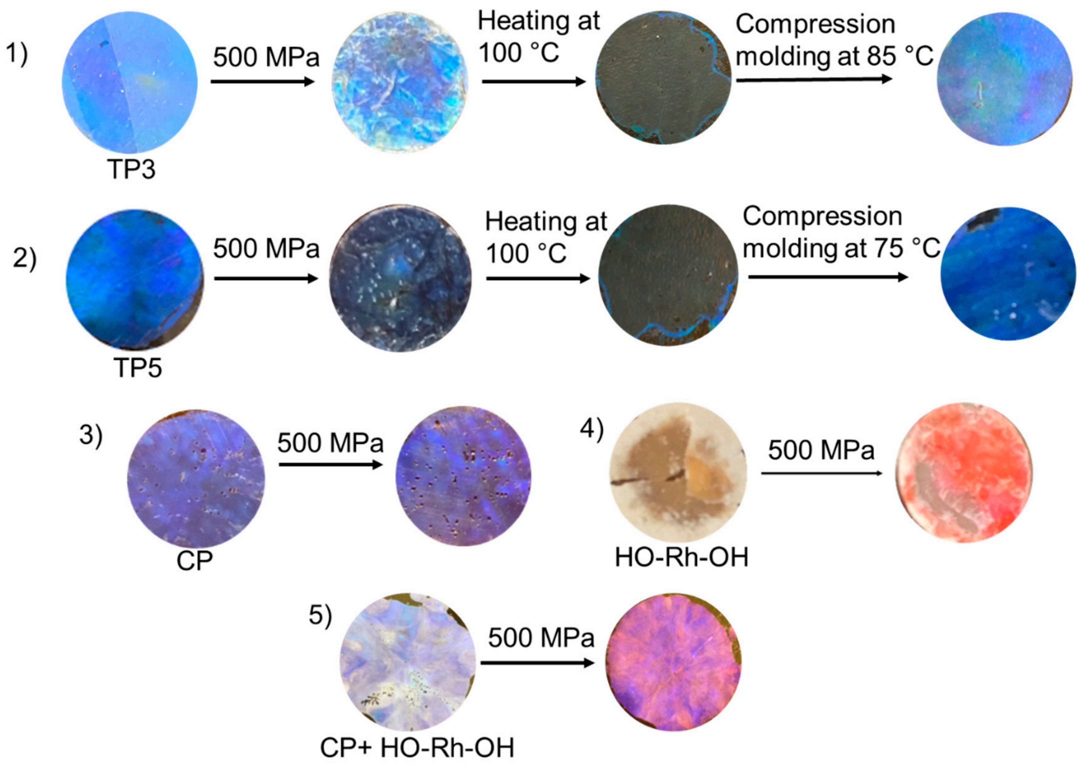

Figure S21. Cholesteric films subjected to 500 MPa pressure, then heated at 100 °C to revert ring-opened **HO-Rh-OH** back to closed ring and compression molding again for testing reproducibility.

1) **TP3** blue colored cholesteric film changes color slightly when subjected to 500 MPa and restores original color on compression molding again

2) **TP5** bright blue colored cholesteric film shows greater color change and changes to a blackish colored film when subjected to 500 MPa, then restores original color on compression molding again.

3) Violet colored cholesteric film of Copolymer (**CP**) that does not have **HO-Rh-OH** shows no color change on subjecting to 500 MPa.

4) Compression molded **HO-Rh-OH** subjected to 500 MPa changes to bright red color but does not show reversibility of changed color.

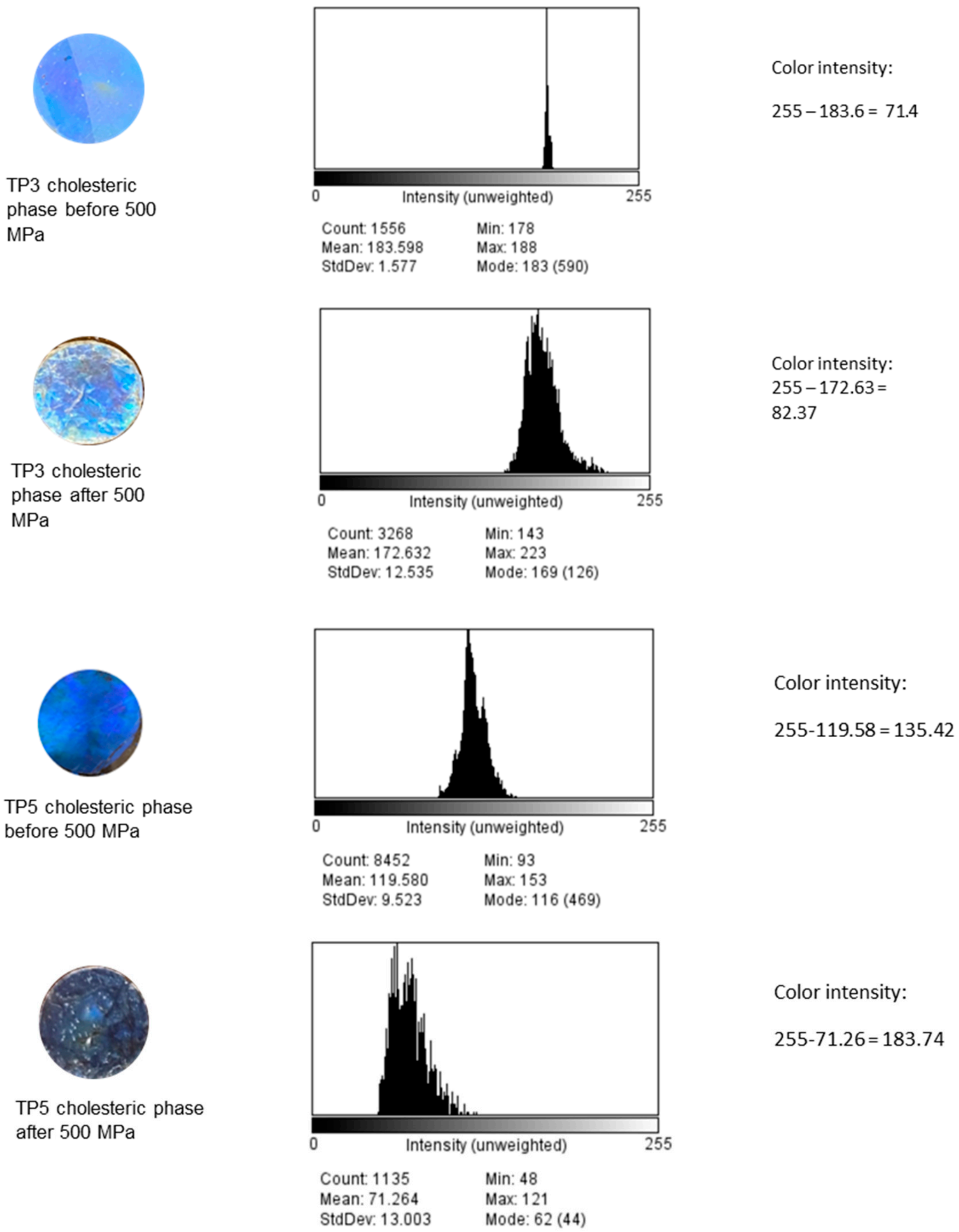

Figure S22. Method used for quantifying colorimetric intensity of cholesteric films of **TP3** and **TP5** before and after 500 MPa using ImageJ software. The value 255 is considered as pure white according to the gray scale. The change in color intensity is larger in **TP5** than **TP3**.

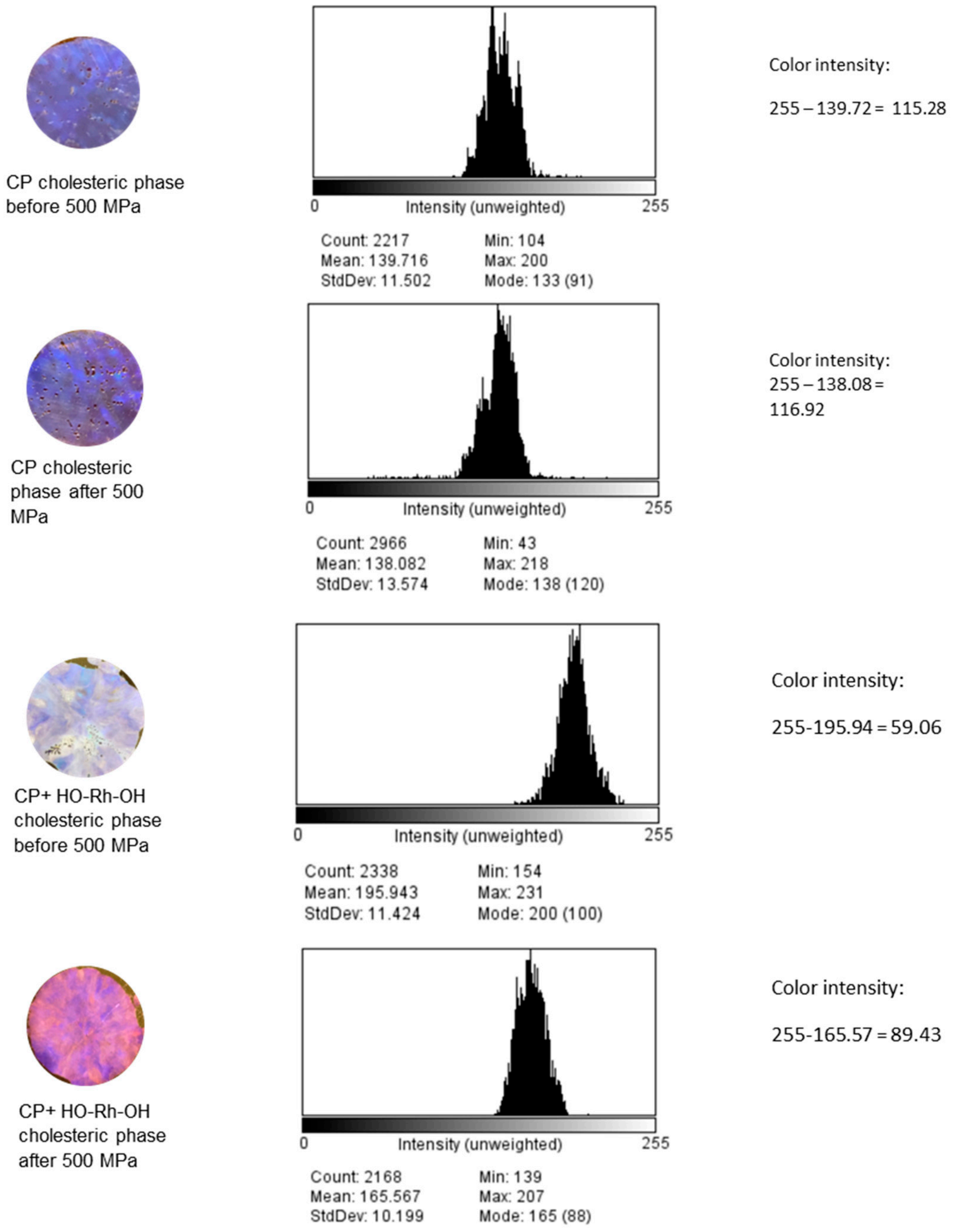

Figure S23. Method used for quantifying colorimetric intensity of cholesteric films of **CP** and **CP+HO-Rh-OH** before and after 500 MPa using ImageJ software. The value 255 is considered as pure white according to the gray scale. The change in color intensity of **CP** is insignificant and change in color intensity of **CP+HO-Rh-OH** were compared with **TP3** and **TP5**.

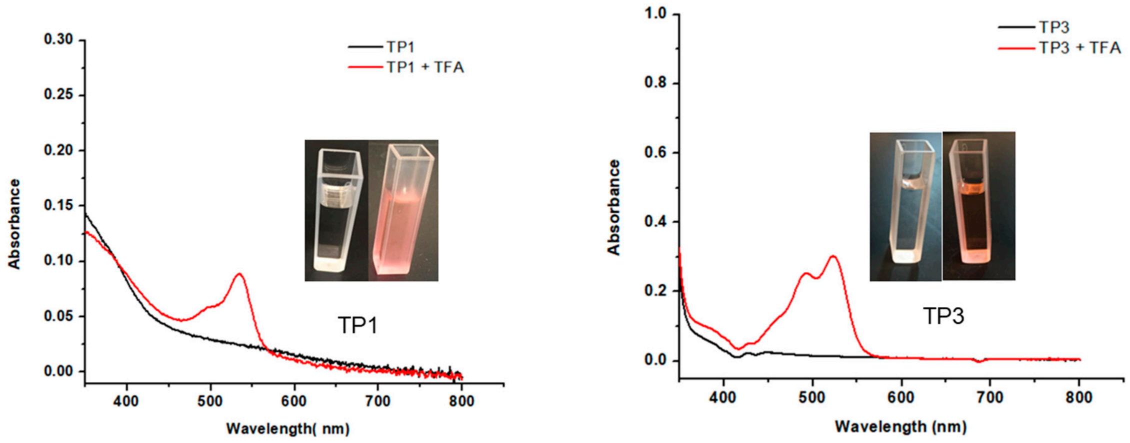

Figure S24. UV-vis absorbance of **TP1** and **TP3** before and after addition of Trifluoroacetic acid

10^{-4} M solutions of **TP1** and **TP3** were prepared in CH_2Cl_2. 10 µL of TFA was added in each of the solutions and the UV-vis absorbance spectra was recorded. A peak at around 535 nm after the addition of TFA indicated the ring-opening reaction of closed ring mechanochromic Rhodamine HO-Rh-OH that confirmed the presence of rhodamine molecule within the monomers and terpolymers. Molar absorptivity of **TP1** = 9×10^2 L/mol cm and **TP3** = 30×10^2 L/mol cm.
